# Survival of pancreatic cancer cells lacking KRAS function

**DOI:** 10.1038/s41467-017-00942-5

**Published:** 2017-10-23

**Authors:** Mandar Deepak Muzumdar, Pan-Yu Chen, Kimberly Judith Dorans, Katherine Minjee Chung, Arjun Bhutkar, Erin Hong, Elisa M. Noll, Martin R. Sprick, Andreas Trumpp, Tyler Jacks

**Affiliations:** 1 David H. Koch Institute for Integrative Cancer Research, Massachusetts Institute of Technology, Cambridge, MA 02139 USA; 2 Dana-Farber Cancer Institute, Boston, MA 02215 USA; 3 Harvard Medical School, Boston, MA 02215 USA; 4 Department of Biology, Massachusetts Institute of Technology, Cambridge, MA 02139 USA; 5 Heidelberg Institute for Stem Cell Technology and Experimental Medicine (HI-STEM), Heidelberg, 69120 Germany; 6 Division of Stem Cells and Cancer, German Cancer Research Center (DKFZ), Heidelberg, 69120 Germany; 7 Howard Hughes Medical Institute, Massachusetts Institute of Technology, Cambridge, MA 02139 USA

## Abstract

Activating mutations in the proto-oncogene *KRAS* are a hallmark of pancreatic ductal adenocarcinoma (PDAC), an aggressive malignancy with few effective therapeutic options. Despite efforts to develop KRAS-targeted drugs, the absolute dependence of PDAC cells on KRAS remains incompletely understood. Here we model complete KRAS inhibition using CRISPR/Cas-mediated genome editing and demonstrate that KRAS is dispensable in a subset of human and mouse PDAC cells. Remarkably, nearly all *KRAS* deficient cells exhibit phosphoinositide 3-kinase (PI3K)-dependent mitogen-activated protein kinase (MAPK) signaling and induced sensitivity to PI3K inhibitors. Furthermore, comparison of gene expression profiles of PDAC cells retaining or lacking *KRAS* reveal a role of KRAS in the suppression of metastasis-related genes. Collectively, these data underscore the potential for PDAC resistance to even the very best KRAS inhibitors and provide insights into mechanisms of response and resistance to KRAS inhibition.

## Abstract (web summary)

Pancreatic cancer cells may develop resistance to KRAS inhibitors due to activation of compensatory pathways. In this study, the authors demonstrate that KRAS is dispensable in a subset of pancreatic cancer and that PI3K signalling may have an important role in mediating tumor growth following KRAS inhibition.

## Introduction

Pancreatic ductal adenocarcinoma (PDAC) is the third leading cause of cancer death in the United States and a major cause of morbidity and mortality worldwide^1, 2^. While advances in combination chemotherapy have improved median survival^3, 4^, long-term survival remains poor^1, 2^, highlighting the need for novel therapeutic approaches.

Genomic studies have identified mutations in the proto-oncogene *KRAS* as a hallmark of PDAC, occurring in >90% of cases^5–8^. KRAS is a small GTPase that acts as a molecular switch to regulate proliferation, differentiation, metabolism, and survival^9^. Oncogenic forms of *KRAS* harboring mutations in codons 12, 13, and 61 are insensitive to GTPase activating protein (GAP)-induced GTP hydrolysis, leading to constitutive activation^10^. Studies in animal models have confirmed an important role of oncogenic *KRAS* in tumor initiation^11^, making KRAS an attractive therapeutic target.

Unfortunately, the development of effective KRAS inhibitors has been hindered by several features of oncogenic KRAS: (1) its high affinity for GTP, impeding the identification of GTP-competitive inhibitors; (2) the difficulty of inducing gain-of-function hydrolytic activity with small molecules; and (3) redundant pathways for membrane localization required for KRAS activity^9, 10^. New approaches to directly inhibit KRAS through covalent binding of specific mutant variants (e.g., G12C)^12, 13^, interference with guanine-exchange factor (GEF) association to prevent initial GTP loading^14, 15^, and destabilization of additional membrane localization complexes^16^ continue to be developed. Furthermore, the success of a recent effort spearheaded by the National Cancer Institute of the United States to develop novel RAS-targeted therapies^17, 18^ requires a better understanding of the dependency of PDAC cells on KRAS as well as predicting resistance mechanisms that could develop in response to KRAS inhibition.

Given the lack of KRAS inhibitors, genetic tools have been used to evaluate the requirement of KRAS in PDAC maintenance. Acute KRAS knockdown by RNA interference (RNAi) decreased cell proliferation and/or induced apoptosis in a series of human PDAC (hPDAC) cancer cell lines^19–21^. Variability in apoptotic response to KRAS knockdown led to the classification of some cells as “KRAS-dependent” and others as “KRAS-independent”^20, 21^. Based on these studies, it was unclear whether the “KRAS-independent” phenotype was a consequence of the incomplete inhibitory effects of RNAi such that residual KRAS protein was sufficient to sustain cell survival and proliferation. Recent evidence for PDAC cell survival in the absence of oncogenic *KRAS* expression derived from a doxycycline (DOX)-inducible oncogenic *KRAS* transgenic mouse model^22^. In this model, DOX treatment led to oncogenic *KRAS* expression in the pancreas to initiate tumorigenesis, while DOX withdrawal halted transgene expression and induced tumor regression. Interestingly, a subset of PDAC tumors recurred lacking *KRAS* transgene expression^22^. Despite these findings, the absolute dependence of PDAC cells on endogenous KRAS, a prerequisite for the successful clinical development of novel KRAS inhibitors, remains unknown.

In this study, we examine the consequence of *KRAS* knockout in PDAC cells using the clustered regularly interspaced short palindromic repeats (CRISPR)/Cas system. The bacterial CRISPR/Cas adaptive immune system, modified for genome editing in mammalian cells, utilizes a single guide RNA (sgRNA) to direct the Cas9 nuclease to cleave matching double-stranded DNA (dsDNA) sequences, resulting in insertions and deletions via error-prone non-homologous end joining repair mechanisms^23^. We confirm the variable dependence of hPDAC cell lines based on prior RNAi studies^20, 21^, and further isolate a subset of hPDAC and murine PDAC (mPDAC) cells that can survive and proliferate despite the absence of endogenous KRAS function. An unbiased chemical screen identifies sensitivity to phosphoinositide 3-kinase (PI3K) inhibition in *KRAS* deficient cells, offering a pharmacologically tractable method to subvert resistance to KRAS blockade. Furthermore, we gain mechanistic insight into how PI3K inhibition simultaneously blocks the mitogen-activated protein kinase (MAPK) and AKT pathways to impair cap-dependent translation and cell viability in the context of *KRAS* ablation. Finally, gene expression profiling defines KRAS-regulated pathways in PDAC cells and reveals KRAS-relevant gene signatures that strongly predict survival in PDAC patients.

## Results

### CRISPR/Cas-mediated *KRAS* knockout in PDAC cells

To evaluate the dependence of PDAC cells on endogenous *KRAS*, we employed CRISPR/Cas technology^23^ to completely eliminate KRAS function. We expressed *Streptococcus pyogenes* Cas9 and a panel of sgRNAs targeting various *KRAS* exons (Supplementary Fig. 1a and Supplementary Table 1) in *KRAS* mutant hPDAC and mPDAC cell lines to identify sgRNAs that effectively induced *KRAS* protein loss (Supplementary Fig. 1b). Given the lack of unique protospacer adhesion motifs (PAM) encompassing mutant codon 12, our sgRNAs did not discriminate between wild-type and mutant forms of *KRAS*, modeling non-selective KRAS inhibition, which comprises the vast majority of approaches currently being evaluated to target KRAS^18^. The consequence of short-term CRISPR/Cas-mediated *KRAS* knockout mimicked the effect of short hairpin RNA (shRNA)-mediated KRAS knockdown on cell viability (Supplementary Fig. 1c, d). Consistent with published results^20^, previously defined “KRAS-independent” cells (8988T, PANC-1) were largely insensitive to sgRNA transduction, while “KRAS-dependent” cells (8902) exhibited significantly decreased viability (Supplementary Fig. 1e).

**Fig. 1:**
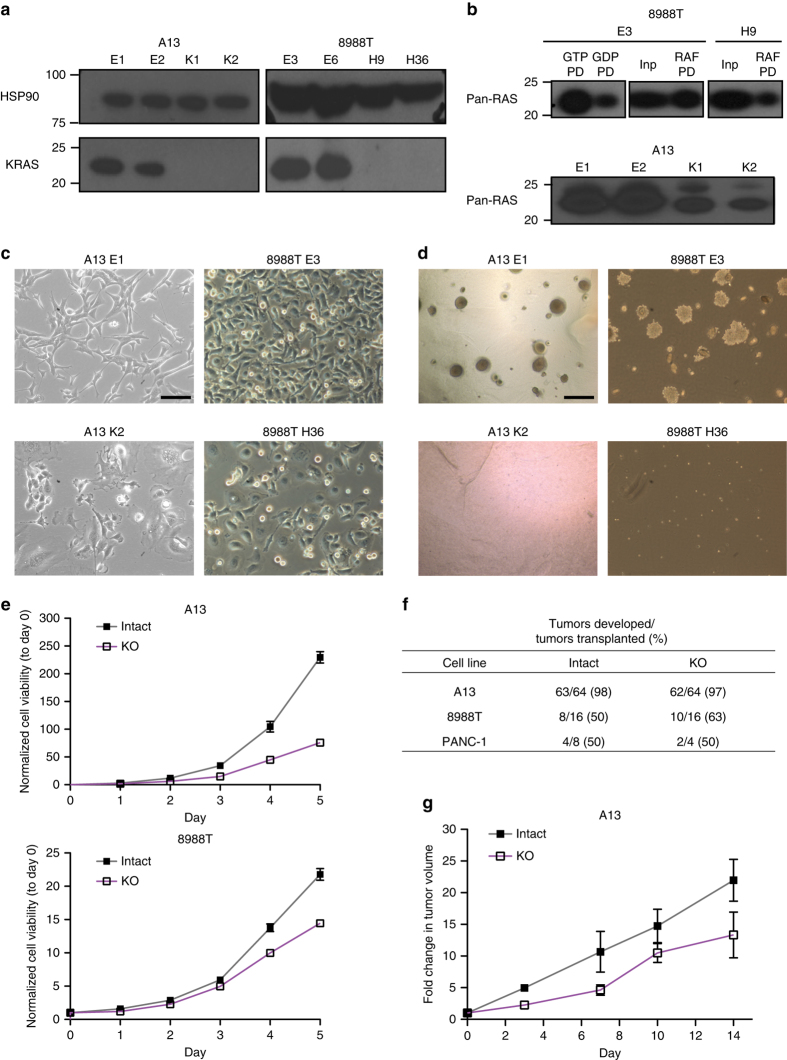
*KRAS* is dispensable for in vitro and in vivo proliferation of PDAC cells. **a** Western blot confirmed loss of KRAS protein in knockout clones (A13-K1,K2, 8988T-H9,H36) compared to intact clones (A13-E1,E2, 8988T E3, E6). HSP90 is loading control. **b** RAS-GTP levels were decreased in knockout (8988T-H9 and A13-K1,K2) compared to intact (8988T E3 and A13-E1,E2) clones. GTPγS (non-hydrolysable)-treated positive control (GTP PD) and GDP-treated negative control (GDP PD) for 8988T E3 are shown. PD pull-down. Inp input before pull-down. **c** *KRAS* deficient clones exhibited altered cell morphology, characterized by increased cell size, cytoplasmic translucency, and smooth edges. Scale bar is 100 µm. **d** *KRAS* deficient clones showed diminished anchorage-independent growth in soft agar. Scale bar is 500 µm. **e** Growth curves for A13 and 8988T *KRAS* intact and deficient (KO) clones. Average cell viability (normalized to day 0) ± s.e.m. is plotted for A13 (*n* = 2 clones) and 8988T (*n* = 4 clones). **f** A13, 8988T, and PANC-1 clones exhibited comparable efficiency generating tumors following subcutaneous transplant in nude mice regardless of *KRAS* status. Shown are cumulative data from two *KRAS* intact and two deficient clones for A13 and 8988T and one intact and one deficient clone for PANC-1. **g** A13 *KRAS* deficient tumors grew at a slower rate than intact tumors. Average tumor volume fold increase (normalized to day 0 when tumors were ~0.5 cm in diameter) ± s.e.m. is plotted (*n* = 8 tumors per group)

We further generated single cell subclones to evaluate whether PDAC cells could survive in the complete absence of KRAS expression. We isolated *KRAS* deficient subclones from 8988T hPDAC and A13 mPDAC cells (Fig. 1a and Supplementary Fig. 2c) and confirmed decreased total RAS activity (RAS-GTP) (Fig. 1b). Sanger sequencing revealed indels in the *KRAS* locus leading to premature stop codons or in-frame indels of important functional domains^24^ (Supplementary Fig. 2a, b), likely perturbing protein folding and stability. PDAC cells that survived *KRAS* loss exhibited perturbations of several growth characteristics. *KRAS* deficient cells displayed altered morphology (Fig. 1c and Supplementary Fig. 2d) comparable to that observed with KRAS knockdown (Supplementary Fig. 1d), significantly diminished anchorage-independent colony formation (Fig. 1d and Supplementary Fig. 2e), and slower proliferation in 2D and 3D culture (Fig. 1e and Supplementary Fig. 2f). Interestingly, *KRAS* deficient cells retained the ability to form subcutaneous tumors in immunocompromised mice (Fig. 1f), though tumors grew more slowly (Fig. 1g).

To ensure that the observed phenotypes were due to on-target *KRAS* mutagenesis, we sequenced the closest exonic sgRNA mismatches and found no off-target mutations in these loci (Supplementary Fig. 3a, b). Furthermore, NRAS and HRAS expression were unaltered, consistent with *KRAS* specificity of the sgRNAs (Supplementary Fig. 3c). Mutation analysis of RNA-sequencing (RNA-Seq) from 8988T (Supplementary Data 1) and A13 (Supplementary Data 2) subclones did not reveal recurrent protein-coding single nucleotide polymorphisms in expressed genes distinguishing all intact and deficient clones. Finally, we re-expressed oncogenic *KRAS* in deficient clones (Supplementary Fig. 3d), and observed a reversal in cell morphology (Supplementary Fig. 3e), in vitro proliferation (Supplementary Fig. 3f), and soft agar colony formation (Supplementary Fig. 3g). Together, these data demonstrate that *KRAS* knockout significantly impacts proliferation in vitro and tumorigenic growth in vivo, confirming the importance of KRAS in PDAC cell maintenance.

### KRAS is dispensable in a subset of PDAC cells

**Fig. 2:**
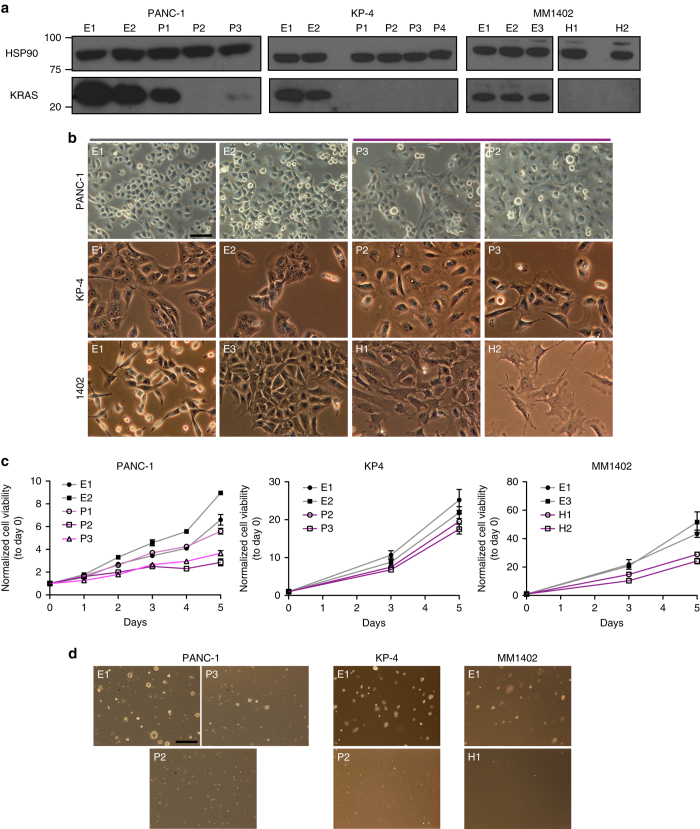
KRAS is dispensable in a subset of PDAC cell lines. **a** Western blot confirmed loss of KRAS protein in knockout clones derived from PANC-1 (P2 complete, P3 partial), KP-4 (P1, P2, P3, P4), and MM1402 (H1, H2) cell lines compared to intact clones (PANC-1-E1,E2; KP-4-E1,E2; MM1402-E1,E2,E3). HSP90 is loading control. **b** *KRAS* deficient clones (purple) exhibited altered cell morphology compared to intact cells (gray). Specific differences include increased cell size, cytoplasmic translucency, and smooth edges. Scale bar is 100 µm. **c** *KRAS* deficient clones showed diminished proliferation in vitro. Average cell viability (normalized to day 0) ± s.e.m. ﻿for each clone is plotted. PANC-1 knockout clone (P2) and partial knockout clone (P3) exhibited a dose-dependent effect of KRAS expression on proliferation compared to intact clones (E1, E2). **d** PANC-1, KP-4, and MM1402 *KRAS* deficient clones showed diminished soft agar colony formation. PANC-1 cells displayed a dose-dependent effect of KRAS expression on anchorage-independent growth. Scale bar is 500 µm

We next evaluated the frequency of KRAS independence in a larger panel of *KRAS* mutant hPDAC and mPDAC cell lines derived from multiple models of *KRAS*-driven murine PDAC (Supplementary Table 2). We successfully isolated *KRAS* deficient clones from the hPDAC cell lines PANC-1 and KP-4 and the mPDAC cell line MM1402 (Fig. 2a). These additional *KRAS* deficient cells exhibited cellular features comparable to those observed in 8988T and A13 knockout cells, including alterations in cell morphology (Fig. 2b), decreased proliferation in vitro (Fig. 2c), and diminished anchorage-independent growth (Fig. 2d). In contrast, we were unable to derive *KRAS* deficient clones from hPDAC cell lines 8902, YAPC, and PSN-1 and mPDAC cell lines D8, E, F, and MM1404, as these cell lines either generated clones that retained KRAS protein (e.g., 8902 and PSN-1, Supplementary Fig. 4a) or did not form recoverable clones at all (e.g., YAPC).

Interestingly, cell lines with lower KRAS protein levels exhibited greater tolerance for *KRAS* knockout (Supplementary Fig. 4b). Recent work has suggested that increased target copy number can permit CRISPR/Cas-mediated lethality in a gene-independent fashion^25^. Indeed, we observed that increased *KRAS* copy number correlated with decreased capacity to generate knockout clones, especially in hPDAC cell lines (Supplementary Fig. 4c). Nonetheless, *KRAS* deficient cells could be isolated from some cell lines (e.g., KP-4 and PANC-1) despite having similarly elevated *KRAS* copy number to cell lines that did not generate knockout clones (e.g., 8902). Together, these data are consistent with prior work^20^ and suggest that KRAS protein levels may be a biomarker of sensitivity to KRAS inhibition in PDAC cells.

Overall, half (3/6) of established hPDAC and one-third (2/6) of primary and established mPDAC cell lines had the capacity to generate *KRAS* deficient clones (Supplementary Table 3), suggesting absolute KRAS independence is not an isolated phenomenon. Moreover, we cannot exclude the possibility that screening additional clones could identify *KRAS* deficient cells even in parental cell lines from which we have been unable to recover knockout clones to date. Nonetheless, endogenous *KRAS* is dispensable in a large fraction of PDAC cells, underscoring the potential for resistance to even the very best KRAS inhibitors.

### *KRAS* knockout cells exhibit PI3K dependence

**Fig. 3:**
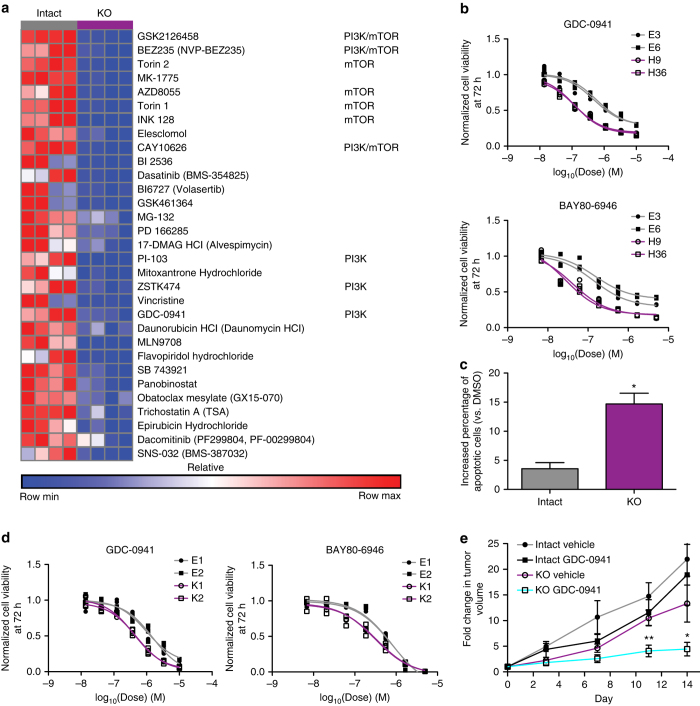
*KRAS* deficient cells are dependent on PI3K. **a** Heat map of area under the curve (AUC) for *KRAS* intact and deficient (KO) clones (columns) treated with various compounds. Row normalized data are presented with red designating high AUC (less sensitive) and blue denoting low AUC (more sensitive). Shown are hit compounds (see “Methods” section) exhibiting greater sensitivity in *KRAS* deficient cells listed in order of ΔAUC from highest to lowest. PI3K and mTOR inhibitors are noted. See Supplementary Data 4 for full data set. **b** Dose-response curves of 8988T *KRAS* intact (gray) and deficient (purple) cells to the pan-PI3K inhibitors GDC-0941 and BAY80-6946. Each replicate (*n* = 3 for each dose) and curve fit are shown. **c** Increased apoptosis (change in percentage Annexin V-positive cells vs. DMSO) in *KRAS* deficient (KO) cells 48 h after 2 μM GDC-0941 treatment. Average ± s.e.m. is plotted (*n* = 2 clones per group). *p < 0.05, two-tailed Student’s *t* test. **d** Dose-response curves of A13 cells to pan-PI3K inhibitors. Each replicate (*n* = 3 for each dose) and curve fit are shown. **e** GDC-0941 significantly decreased the growth rate of *KRAS* deficient (KO) but not intact A13 transplanted tumors in nude mice. Average tumor volume fold increase (normalized to start of treatment at day 0) ± s.e.m. (*n* = 8 tumors per group) is plotted. *p < 0.05, **p < 0.01, two-tailed Student’s *t* test for measurements at each time point comparing GDC-0941 to vehicle

We next sought to elucidate the mechanisms that permit the proliferation and survival of PDAC cells in the absence of KRAS. While previous work reported that marked overexpression of YAP1 could support the growth of a subset of PDAC tumors following loss of KRAS expression^22, 26^, we did not observe similar elevations in YAP1 protein levels in *KRAS* deficient compared to intact cells (Supplementary Fig. 5a). Moreover, *KRAS* deficient cells did not exhibit increased sensitivity to verteporfin, a YAP–TEAD interaction inhibitor^27^, or to YAP1 knockdown/knockout compared to intact cells (Supplementary Fig. 5b–f). Therefore, we employed high-throughput drug screening to identify unique dependencies in *KRAS* deficient cells. We screened 8988T *KRAS* intact and deficient clones against a compound library comprised of kinase inhibitors, epigenetic modifiers, and chemotherapeutic agents, many of which are being tested in clinical trials or are FDA approved (Supplementary Data 3). While no compound selectively impaired the viability of *KRAS* intact cells, deficient cells exhibited increased sensitivity to pan-PI3K and mTOR inhibitors (Fig. 3a and Supplementary Data 4). As dose-response curves and direct observation suggested a cytotoxic effect of pan-PI3K inhibitors rather than the cytostatic effect of mTOR inhibition (Supplementary Fig. 6), we chose to further characterize the consequence of PI3K inhibition on *KRAS* deficient cells.

**Fig. 4:**
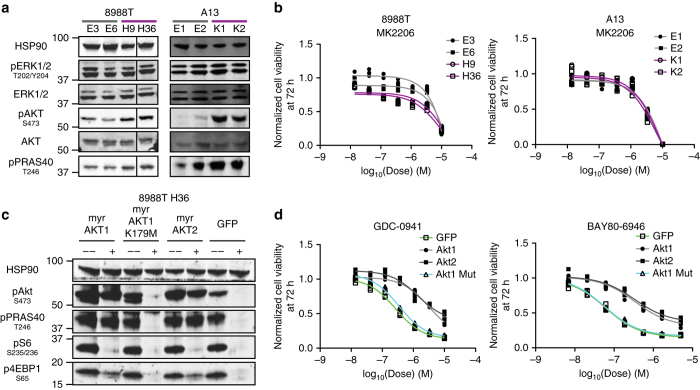
PI3K inhibition functions through AKT-dependent and -independent mechanisms. **a** Western blot showed stable pERK1/2 but increased pAKT and pPRAS40 levels in 8988T and A13 *KRAS* deficient (purple) cells, consistent with PI3K/AKT pathway activation. HSP90 is loading control. **b** Dose-response curves of 8988T and A13 *KRAS* intact (gray) and deficient (purples) clones to the pan-AKT inhibitor MK2206. Each replicate (*n* = 3 for each dose) and curve fit are shown. **c** Western blot showed sustained phosphorylation of AKT and downstream targets (PRAS40, S6, and 4EBP1) only in *myr-AKT1-* and *myr-AKT2-*expressing cells but not in *myr-AKT1 (K179M)-* or control *GFP*-expressing cells following 4 h of 2 μM GDC-0941 treatment. **d** Dose-response curves of cell lines in **c** treated with GDC-0941 and BAY80-6946 demonstrated a marked decrease in PI3K sensitivity with *myr-AKT1* or *myr-AKT2* overexpression. Each replicate (*n* = 3 for each dose) and curve fit are shown

We confirmed enhanced sensitivity to the pan-PI3K inhibitors GDC-0941 and BAY80-6946 in additional 8988T *KRAS* deficient clones (Fig. 3b and Supplementary Fig. 7a) and that GDC-0941 increased apoptosis in deficient cells (Fig. 3c). In addition, we found that A13 *KRAS* deficient clones were more sensitive to PI3K inhibition than their intact counterparts both in vitro and in vivo (Fig. 3d, e). Finally, KP-4 and MM1402, but not PANC-1, knockout clones demonstrated increased sensitivity to GDC-0941 (Supplementary Fig. 7a). Treatment of 8988T and A13 clones with combinations of PI3K class I isoform-specific inhibitors revealed a synergistic effect of p110α inhibition with p110β- or p110δ-specific inhibitors (Supplementary Fig. 7b, c), suggesting the need for pan-class I PI3K inhibition for full effect. Biochemically, we observed stable MAPK phosphorylation but significantly increased PI3K/AKT pathway activation in 8988T and A13 deficient cells (Fig. 4a and Supplementary Fig. 7d, e). Interestingly, MM1402 and PANC-1, but not KP-4, knockout cells also showed increased pAKT levels at steady state (Supplementary Fig. 7f). These data suggest that PI3K/AKT hyperactivation and PI3K inhibitor sensitivity are features of most PDAC cells following *KRAS* knockout.

We next explored the mechanisms underlying PI3K/AKT activation in 8988T and A13 *KRAS* deficient cells. As activating PI3K pathway mutations (Supplementary Data 1 and 2) and changes in the protein levels of the phosphoinositide phosphatases PTEN and INPP4B (Supplementary Fig. 8a) were not observed in deficient cells, we hypothesized that feedback stimulation of PI3K by upregulated receptor tyrosine kinases (RTKs)^28–30^ could be occurring. While we observed PDGFRβ and FGFR2 upregulation in *KRAS* deficient cells (Supplementary Fig. 8b), inhibition of PDGFR and FGFR alone or in combination did not show a greater effect on the proliferation of deficient cells (Supplementary Fig. 8c). Similarly, RTK array profiling of A13 intact and deficient clones did not reveal significant differences in phosphorylation across a broader array of RTKs (Supplementary Fig. 8d). In contrast, stimulation with any of the RTK ligands EGF, PDGF-BB, or FGF1 decreased sensitivity of deficient cells to PI3K inhibition (Supplementary Fig. 8e, f). Together, these data suggest that individual RTKs may be sufficient but not necessary to support PI3K activation in *KRAS* deficient cells, indicating compensatory mechanisms. Nonetheless, PI3K represents a convergent node in PDAC cells lacking KRAS function.

### Simultaneous MAPK and AKT blockade by PI3K inhibition

To dissect the mechanism underlying PI3K inhibitor sensitivity in *KRAS* deficient cells, we evaluated the effect of the pan-AKT inhibitor MK2206 on cell viability in vitro. Surprisingly, AKT inhibition did not recapitulate the differential sensitivity observed with PI3K inhibition in 8988T and A13 cells (Fig. 4b). In contrast, overexpression of constitutively active myristoylated (*myr*) forms of *AKT1* or *AKT2* (but not the kinase-dead mutant *AKT1* (*K179M*)) prevented GDC-0941-induced AKT pathway inhibition and markedly decreased PI3K inhibitor sensitivity (Fig. 4c, d and Supplementary Fig. 9a–d). Overall, PI3K inhibitor-mediated AKT blockade is necessary but insufficient for its effect on cell viability.

**Fig. 5:**
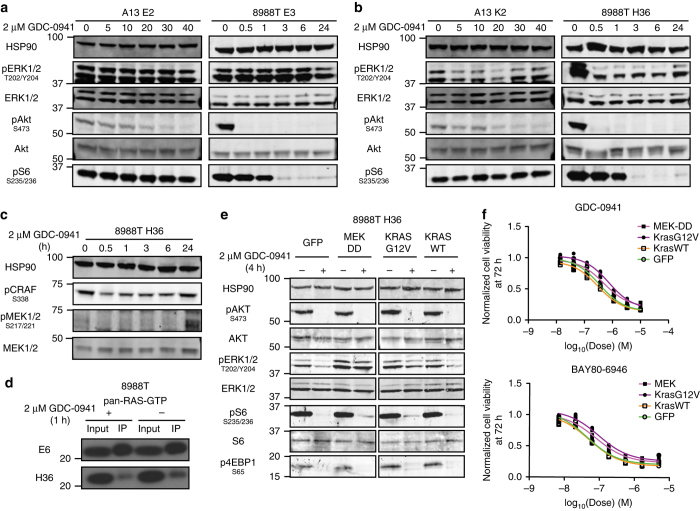
MAPK blockade following PI3K inhibition in *KRAS* deficient cells. **a** Western blot showed no change in pERK1/2 levels in *KRAS* intact cells at designated times (minutes for A13, hours for 8988T) following GDC-0941 treatment. HSP90 is loading control. **b** Western blot demonstrated a transient decrease in pERK1/2 levels in *KRAS* deficient cells at designated times (minutes for A13, hours for 8988T) following GDC-0941 treatment. **c** Western blot showed a transient decrease in phosphorylation of the MAPK pathway regulators CRAF and MEK1/2 following GDC-0941 treatment in *KRAS* deficient cells. **d** Western blot of RAS-GTP levels in *KRAS* intact (E6) and deficient (H36) clones following 1-h treatment with GDC-0941 showed a specific decline in deficient cells. **e** Overexpression of constitutively active MEK (*MEK-DD*) or oncogenic *KRAS*-*G12V*, but not *KRAS-WT* or *GFP*, blocked pERK1/2 inhibition by a 4-h treatment with GDC-0941. **f** *MEK-DD* and *KRAS-G12V*-transduced cells from **e** showed decreased sensitivity to PI3K inhibition compared to control *GFP-* and *KRAS-WT*-transduced cells

Recent work indicated that PI3K inhibitors inactivate the MAPK pathway in cells harboring PI3K pathway mutations to induce apoptosis^31, 32^. Indeed, while GDC-0941 sustainably suppressed pAKT in both *KRAS* intact and deficient cells, a transient acute decrease in pERK1/2 levels lasting minutes to hours only occurred in deficient cells (Fig. 5a, b and Supplementary Fig. 10a, b). Surprisingly, the effect of GDC-0941 appeared to be due to inhibition of wild-type RAS activity upstream of the MAPK pathway, as RAS-GTP, pCRAF, and pMEK1/2 levels were diminished in *KRAS* deficient cells (Fig. 5c, d). Consistent with this observation, GDC-0941 also induced a decline in pERK1/2 levels in *KRAS* wild-type hPDAC BxPC3 and 293 human embryonic kidney cells (Supplementary Fig. 10c).

Several lines of evidence support the hypothesis that simultaneous MAPK and AKT inhibition by GDC-0941 underlie its therapeutic effect in the absence of oncogenic *KRAS*. First, in PANC-1 *KRAS* deficient cells, which do not exhibit enhanced PI3K inhibitor sensitivity, GDC-0941 effectively suppressed phosphorylation of AKT without inducing a decrease in pERK1/2 levels (Supplementary Fig. 10c). Second, the MEK inhibitor AZD6244 synergized with MK2206 in both *KRAS* intact and deficient 8988T and A13 cells (Supplementary Fig. 10d). However, while AZD6244 enhanced the effect of GDC-0941 on *KRAS* intact cells, such synergy was absent in deficient cells (Supplementary Fig. 10e), likely due to the MAPK inhibitory effect already provided by GDC-0941. Finally, overexpression of constitutively active MEK (*MEK-DD*) or sgRNA-resistant oncogenic *KRAS* (but not wild-type *KRAS*) prevented pERK1/2 decline and reduced PI3K inhibitor sensitivity in deficient cells (Fig. 5e, f and Supplementary Fig. 10f, g).

**Fig. 6:**
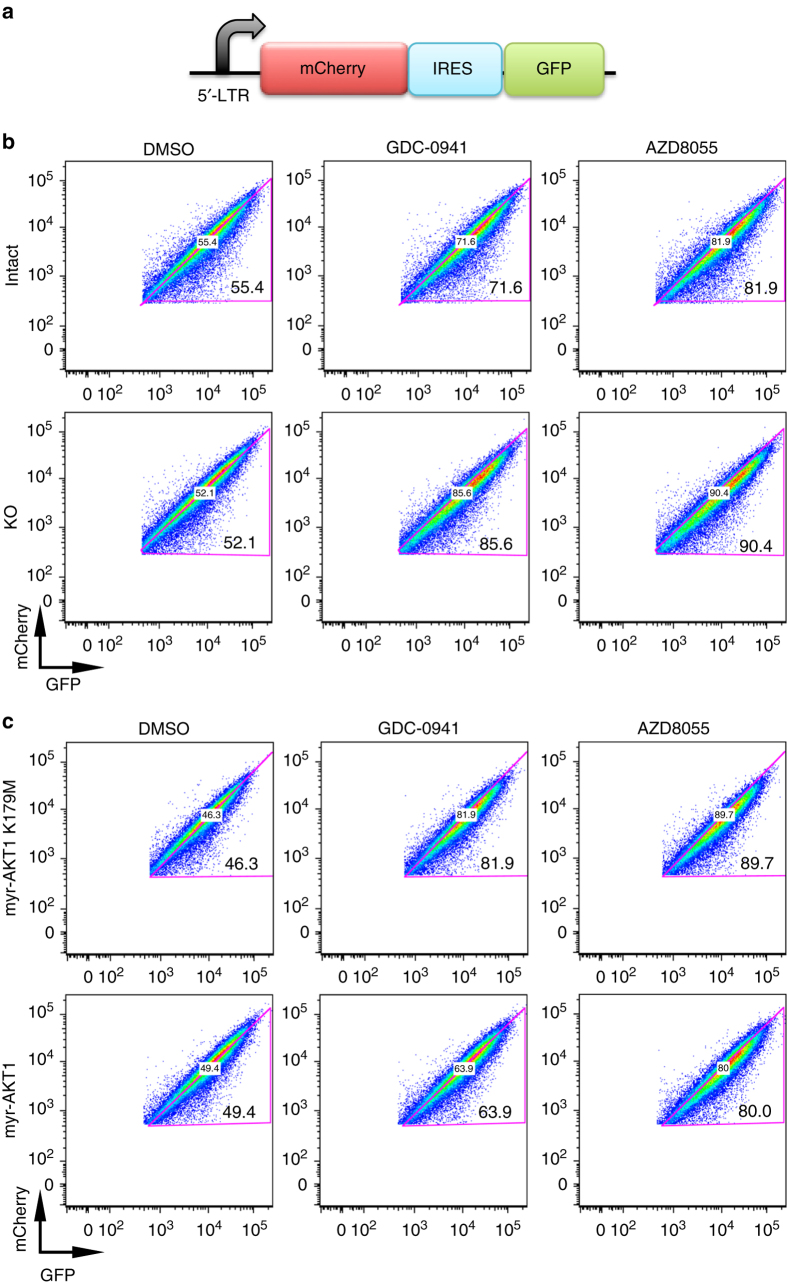
PI3K inhibition enhances cap-dependent translation inhibition in *KRAS* deficient cells. **a** Schematic of cap-dependent translation reporter construct. 5′-LTR 5′ long terminal repeat of MSCV virus with promoter activity. In transduced cells, mCherry expression correlates with cap-dependent translation and GFP expression correlates with cap-independent translation initiated via an internal ribosomal entry site (IRES). **b** FACS plots of GFP and mCherry fluorescence in *KRAS* intact and deficient (KO) cells. *KRAS* deficient cells exhibited a greater decrease in mCherry (relative to GFP) expression when treated for 24 h with GDC-0941 (2 μM) or the mTORC1/2 inhibitor AZD8055 (100 nM) than intact cells. Triangle gates were drawn along the midline diagonal of the FACS plots of DMSO-treated cells and maintained in plots of drug treatment. Numbers denote percentages of cells within gate and is inversely related to cap-dependent translation of reporter. **c** FACS plots of GFP and mCherry fluorescence in *KRAS* deficient cells transduced with *myr-AKT1* or *myr-AKT1 (K179M)*. Wild-type *AKT1* expression decreased the effect of GDC-0941 on cap-dependent translation compared to its kinase-dead variant

Prior research has suggested that the MAPK and AKT pathways may converge on the 4EBP1-EIF4E axis to regulate cap-dependent translation in cancer cells^33, 34^. Interestingly, oncogenic gene signatures associated with the cap-dependent translation mediators MYC and EIF4E^35, 36^ were enriched in gene expression analysis of 8988T *KRAS* intact cells (MSigDB/GSEA, Supplementary Data 11), suggesting that *KRAS* may also regulate this process in this cell line. While we did not observe a difference in MYC protein levels (Supplementary Fig. 8a), baseline phospho-4EBP1 levels were reduced in 8988T *KRAS* deficient cells (Supplementary Fig. 7d), permitting enhanced 4EBP1 sequestration of EIF4E to restrain cap-dependent translation. Furthermore, *KRAS* deficient cells exhibited greater sensitivity to inhibitors of mTOR (Supplementary Fig. 6b), an important upstream regulator of translational control mediated through 4EBP1 phosphorylation. Therefore, we hypothesized that GDC-0941 treatment might further decrease 4EBP1 phosphorylation in these *KRAS* deficient cells, pushing them beyond a threshold to functionally impair cap-dependent translation. To test this, we transduced 8988T *KRAS* intact and deficient cells with an *mCherry-IRES-GFP* translation reporter (Fig. 6a). *KRAS* deficient cells exhibited a more marked decrease in cap-dependent translation when treated with GDC-0941 or the mTORC1/2 inhibitor AZD8055 than intact cells (Fig. 6b). Moreover, the effects of GDC-0941 on cell viability, 4EBP1 phosphorylation, and cap-dependent translation were rescued by overexpression of *myr-AKT1* but not *myr-AKT1 K179M* (Figs. 4c, d and 6c and Supplementary Fig. 9c, d). Taken together, cap-dependent translation may be a node of MAPK and AKT convergence, which underlies the sensitivity to PI3K inhibition in *KRAS* deficient cells.

### Combined KRAS and PI3K inhibition as a therapeutic approach

**Fig. 7:**
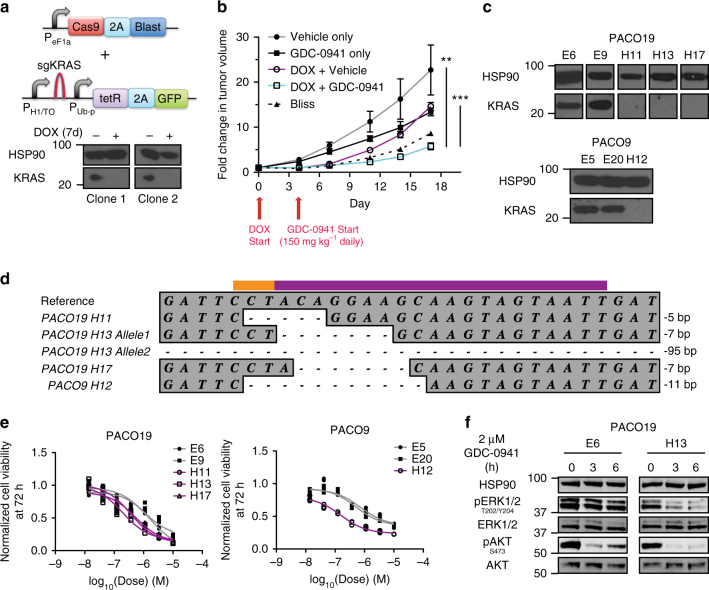
Combined KRAS and PI3K inhibition as a therapeutic approach in PDAC cells. **a** Schematic of lentiviral constructs to express Cas9 and a doxycycline (DOX)-inducible sgRNA targeting *KRAS* (sgKRAS). P_eF1a_ ubiquitously expressed elongation factor 1a promoter. 2A self-cleaving peptide. Blast blasticidin resistance gene. TRE tetracycline-responsive element. P_H1/TO_ H1 promoter with Tet-operator sites. P_Ub-P_ ubiquitin promoter. tetR Tet-repressor. DOX treatment relieves tetR repression of H1 promoter to permit sgKRAS expression. Western blot showed complete KRAS protein ablation in two different A13 *mmKras*.*366* clones after 7 days of DOX treatment in vitro. **b** Combined KRAS (by DOX-inducible *mmKras*.*366*) and PI3K (by GDC-0941) inhibition in established subcutaneous tumors effectively inhibited tumor growth, whereas inhibition of KRAS or PI3K alone was insufficient to suppress tumor growth long term. Average tumor volume fold increase (normalized to start of DOX treatment, pooled for both clones in **a**) ± s.e.m. (*n* = 10 tumors per group) is plotted. Dashed line denotes bliss independence for additive effect of DOX and GDC-0941, consistent with synergism starting at day 11. **p < 0.01, two-tailed Student’s *t* test, DOX + GDC-0941 vs. Vehicle only at end of experiment. ***p < 0.001, two-tailed Student’s *t* test, DOX + GDC-0941 vs. DOX + Vehicle at end of experiment. **c** Western blot confirmed loss of KRAS protein in knockout clones derived from PACO19 (H11, H13, H17) and PACO9 (H12) compared to intact clones (PACO19-E6,E9; PACO9-E5,E20). HSP90 is loading control. **d** *KRAS* alleles from PACO19 and PACO9 clones showed out-of-frame indels in *KRAS* deficient clones. Reference corresponds to UCSC hg19 sequence. All clones retained a single indel allele except for PACO19 H13 for which two different indels were identified, one of which was a 95 bp deletion that is not pictured in full. The purple and orange bars denote the sgRNA and PAM sequences, respectively. **e** Dose-response curves of PACO19 and PACO9 *KRAS* intact (gray) and deficient (purple) clones to the pan-PI3K inhibitor GDC-0941. Each replicate (*n* = 3 for each dose) and curve fit are shown. **f** Western blot showed a decrease in pERK1/2 levels in *KRAS* deficient (H13) but not intact (E6) clones derived from PACO19 cells following GDC-0941 treatment

In order to recapitulate the therapeutic effect of combined KRAS and PI3K inhibition in established PDAC tumors in vivo, we engineered A13 cells to stably express Cas9 and a DOX-inducible *KRAS*-targeting sgRNA (*mmKras*.*366*) and generated clones that efficiently ablated KRAS protein expression following DOX treatment in vitro (Fig. 7a). To account for clonal and animal differences, we subcutaneously transplanted two clones, one on each flank, in immunocompromised mice. Notably, there was minimal variation in tumor growth rate between the two clones. Following tumor establishment, administration of DOX feed led to *mmKras.366* expression in established tumors and acute suppression of tumor growth (Fig. 7b). Importantly, KRAS inhibition alone was insufficient to maintain tumor growth suppression, possibly due to selection of escapers harboring non-frameshift mutations and/or cells that bypassed the requirement of KRAS by PI3K/AKT activation. Supporting the latter hypothesis, subsequent treatment with GDC-0941 more markedly suppressed the growth of *KRAS* knockout tumors (Fig. 7b).

Western blotting of tumors confirmed decreased KRAS protein levels following DOX administration (Supplementary Fig. 11a, b) and on-target inhibition of AKT phosphorylation with GDC-0941 treatment (Supplementary Fig. 11a, c). As with in vitro knockout, we saw increased phosphorylation of AKT with in vivo *KRAS* knockout (Supplementary Fig. 11a, c). Interestingly, we observed an increase in KRAS protein levels in DOX-treated mice also treated with GDC-0941 compared to vehicle (Supplementary Fig. 11b), suggesting selective depletion of *KRAS* knockout cells and outgrowth of technical escapers that did not efficiently induce loss-of-function mutations in *KRAS*. To evaluate for this directly, we performed massively parallel sequencing to analyze allelic fractions of the *KRAS* locus from these tumors (Supplementary Fig. 11d, e) with the hypothesis that there would be an increase in the proportion of non-frameshift (NFS) mutant reads (protein retained) compared to frameshift (FS) reads (protein loss) in tumors subject to combined DOX and GDC-0941 treatment. In pairwise comparisons of DOX + GDC-0941 tumors (*n* = 6) with each of the DOX tumors (*n* = 6), the NFS mutant read fraction was found to be significantly enriched in the DOX + GDC samples in 72% (26/36) of all comparisons (*χ* ^2^-test of proportions, *p* < 0.05). Collectively, these data demonstrate that PI3K inhibition synergizes with acute *KRAS* inhibition in vivo, highlighting that this combination may be a viable therapeutic strategy in established PDAC tumors.

To further evaluate combined KRAS and PI3K inhibition as a therapeutic strategy in a clinically relevant system, we utilized CRISPR/Cas-mediated genome editing to generate *KRAS* deficient clones from low-passage primary patient-derived *KRAS* mutant hPDAC cell lines. Importantly, we were able to isolate deficient clones from both cell lines analyzed (Fig. 7c, d and Supplementary Tables 2 and 3). Similar to established hPDAC cell lines, *KRAS* deficient clones derived from primary hPDAC cell lines demonstrated enhanced sensitivity to PI3K inhibition (Fig. 7e) and combined MAPK and AKT blockade following pharmacological PI3K inhibition (Fig. 7f). Together, these data support the synergy of KRAS and PI3K inhibition in a variety of in vitro and in vivo PDAC models.

### Identification of KRAS-regulated pathways in PDAC cells

**Fig. 8:**
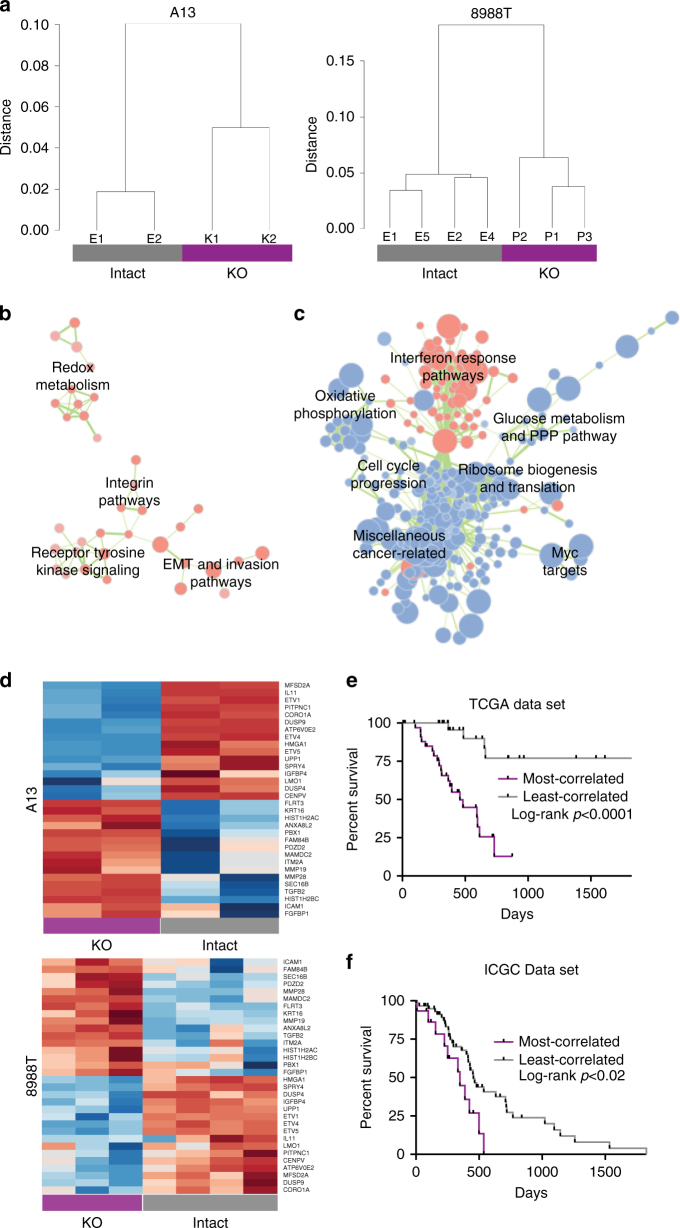
Multiple dysregulated cellular processes in *KRAS* deficient cells. **a** Unsupervised hierarchical clustering dendrograms of 8988T and A13 clones indicated clean segregation between *KRAS* intact and deficient (KO) cells within each cell line. **b** Network representation of overlapping enriched GSEA/MSigDB gene sets in the A13 knockout signature (*p* < 0.05, FDR < 0.25). Each circle represents a gene set with circle size corresponding to gene set size and intensity corresponding to enrichment significance. Red is upregulated and blue is downregulated. Each line corresponds to minimum 50% mutual overlap with line thickness corresponding to degree of overlap. Cellular processes associated with related gene sets are listed. **c** Network representation of overlapping enriched GSEA/MSigDB gene sets in the 8988T knockout signature (*p* < 0.005, FDR < 0.1). **d** Heat map of a 32-gene combined knockout signature generated through ICA analysis of A13 and 8988T gene expression data sets. Gene names are listed in rows. Row normalized gene expression values are shown where red designates relative upregulation and blue designates relative downregulation. **e** Kaplan–Meier plots of survival of human patients from the TCGA PDAC cohort whose tumors most correlated (top quintile) and least correlated (bottom quintile) with the combined knockout signature in **d** (UP genes only). Log-rank (Mantel–Cox) *p* value is shown. **f** Kaplan–Meier plots of survival of human patients from the ICGC cohort whose tumors most correlated (top quintile) and least correlated (remaining tumors) with the combined knockout signature in **d** (UP genes only). Log-rank (Mantel–Cox) *p* value is shown

In addition to elucidating mechanisms of resistance to KRAS inhibition, we uncovered key biological processes regulated by KRAS in PDAC cells by comparing the gene expression profiles of *KRAS* intact and deficient cells. Specifically, we performed RNA-Seq on multiple 8988T and A13 clones. Unsupervised hierarchical clustering cleanly segregated intact from knockout clones (Fig. 8a), and pairwise differential expression analysis identified a large number of genes with significantly altered expression (Supplementary Data 5 and 6). We performed independent component analysis (ICA), a blind source separation approach (see “Methods” section), to generate high-resolution gene signatures associated with *KRAS* knockout (Supplementary Fig. 12a, b and Supplementary Data 7 and 8). To gain insight into the knockout signature, we performed gene set enrichment analysis (GSEA)^37, 38^ across gene expression data sets in MSigDB^38^. As internal validation of our analysis, GSEA revealed anti-correlation of the knockout signatures with genes upregulated by expression of oncogenic *KRAS* in primary epithelial cells (Supplementary Data 9–12). We further compared our gene signatures to data sets generated using the DOX-inducible *KRAS* transgene mouse model to modulate KRAS levels^22, 39, 40^. Given the high degree of heterogeneity observed between tumors and conditions in these data sets, we used ICA (Supplementary Data 13) to identify KRAS-ON and KRAS-OFF signatures associated with acute (24-h) KRAS withdrawal^8^ (Supplementary Fig. 12c), surviving cells following longer-term KRAS withdrawal^13^ (Supplementary Fig. 12d), and KRAS-independent relapsed tumors in mice^9^ (Supplementary Fig. 13a). The KRAS-OFF signatures from these data sets were strongly enriched in our knockout signatures (Supplementary Fig. 13b), supporting the robustness of our *KRAS* knockout signatures across species and model systems.

We next examined GSEA results to identify key pathways regulated by KRAS in PDAC cells (Supplementary Data 14–17). A13 knockout cells showed statistically significant enrichment of a limited number of curated gene sets in pathways previously associated with KRAS, including EMT, integrin, and receptor tyrosine-kinase (RTK) signaling, and redox metabolism^20, 41, 42^ (Fig. 8b). In contrast, 8988T cells exhibited alterations in a large number of biologic processes (Fig. 8c). Consistent with cellular phenotypes, 8988T knockout cells showed decreased expression of genes related to cell cycle progression, nucleotide metabolism, and oxidative phosphorylation (Fig. 8c). Importantly, we uncovered novel gene expression changes associated with KRAS function, including alterations in the expression of genes associated with ribosomal biogenesis, protein translation, and interferon response genes.

### KRAS-relevant signatures predict survival in PDAC patients

**Fig. 9:**
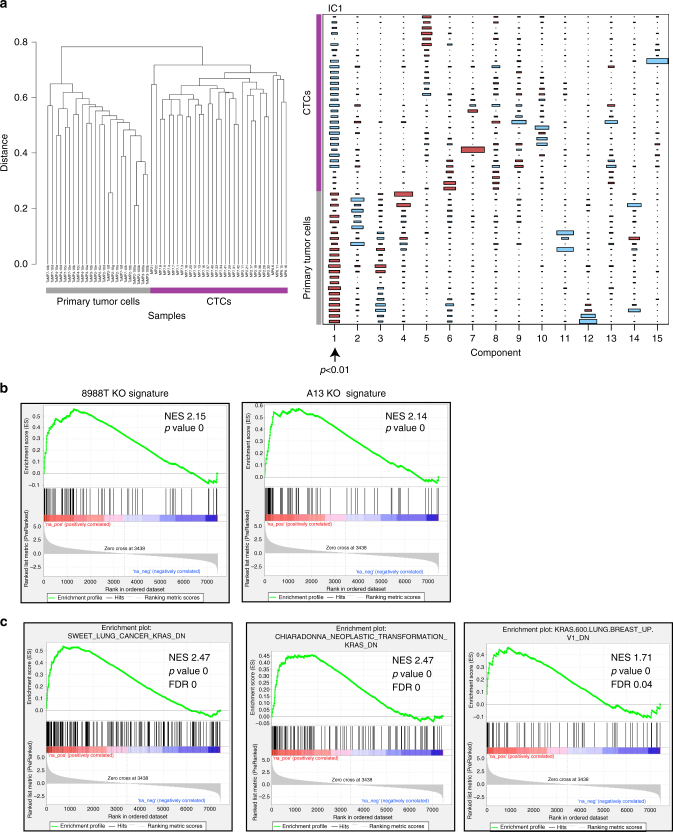
*KRAS* knockout signatures correlate with CTC gene expression. **a** Unsupervised hierarchical clustering and ICA analysis of single cell RNA-Seq data from Ting et al.^44^ using circulating tumor cells (CTCs) and primary tumor cells from a *Kras;p53* mutant mouse model. ICA analysis identified a signature (component 1 (IC1) in Hinton diagram) that distinguished CTCs from primary tumors (*p* < 0.01, Mann–Whitney *U*-test). **b** GSEA plots of 8988T and A13 knockout signatures (top/bottom 2% genes) enriched in ICA-derived CTC signature shown above. Normalized enrichment scores (NES) and *p* values are listed for each gene set. **c** Significantly enriched MSigDB gene sets in CTCs include genes downregulated (DN) following KRAS expression in the *KrasLA2* lung cancer mouse model (Sweet), mouse fibroblasts (Chiaradonna), and in primary human lung and breast epithelial cells (KRAS.600). NES, *p* values, and FDR are listed for each gene set

Recent data from mouse models demonstrated an inverse relationship between proliferation and metastatic capacity in PDAC^43^. Interestingly, we observed a similar inverse relationship in the expression of genes associated with cell proliferation (cell cycle progression, nucleotide metabolism, and protein translation) and those associated with the metastatic process (EMT, invasion, and integrin pathways) based on *KRAS* status (Fig. 8b, c). Moreover, our knockout signatures were strongly enriched in gene expression signatures derived from circulating tumor cells (CTCs) (compared to primary tumors) in a *Kras;p53* mutant PDAC model^44^ (Fig. 9a, b). Therefore, we hypothesized that the *KRAS* knockout signatures may predict worse patient survival.

We analyzed data from The Cancer Genome Atlas (TCGA) and ranked early stage (mostly resected) primary human PDAC tumors based on gene expression correlation to the knockout signatures (Supplementary Data 18). As predicted, we observed that tumors highly correlated with either the 8988T or A13 knockout signatures were associated with poor patient survival (Supplementary Fig. 14a). The tumors most correlated with the knockout signatures expressed genes associated with EMT, invasion, focal adhesion/integrin signaling, and metastasis (Supplementary Fig. 14b, c). Despite their poor prognosis, these tumors showed decreased expression of ribosomal biogenesis, protein translation, oxidative phosphorylation, and cell cycle progression genes (Supplementary Fig. 14b, d). In these survival analyses, we noticed that tumors most correlated with the 8988T or A13 knockout signatures had non-significant overlap (*p* = 0.44, hypergeometric test). In contrast, tumors least correlated with the knockout signatures were largely the same regardless of the cell line from which the signature was derived (*p* = 1.04 × 10^−7^, hypergeometric test). This suggested that loss of oncogenic KRAS function leads to the de-repression of genes that promote a more aggressive phenotype. Consistent with this hypothesis, the murine CTC signature exhibited enrichment of gene sets downregulated by oncogenic *KRAS* expression in various cell types (Supplementary Fig. 9c).

We next integrated our hPDAC and mPDAC gene expression profiles to define a core set of KRAS-regulated genes with greater prognostic value. We jointly analyzed the 8988T and A13 signatures to generate a 32-gene knockout signature (see “Methods” section, Fig. 8d). While the downregulated genes in this set related to MAPK signaling (*DUSP4*, *DUSP9*, *SPRY4*, *ETV1*, *ETV4*, *ETV5*), the upregulated genes supported pathways involved in the metastatic cascade, including EMT (*TGFB2*, *PBX1*, *FGFBP1*), cell adhesion (*FLRT3*, *ICAM1*), and extracellular matrix breakdown (*MMP19*, *MMP28*). Strikingly, ranking tumors by expression of just the 16 upregulated genes was sufficient to improve prediction of PDAC patient survival in the TCGA cohort in multivariable analyses (Fig. 9e, Supplementary Fig. 14e, and Supplementary Data 18). We confirmed the prognostic capability of this 16-gene signature for survival in a separate PDAC patient cohort from the International Cancer Genome Consortium (ICGC)^45^ (Fig. 8f and Supplementary Table 4). Collectively, these data offer an independent prognostic gene signature to predict survival in early stage patients with PDAC and implicate loss of KRAS-related transcriptional suppression as a potential mechanism toward PDAC metastasis.

**Fig. 10:**
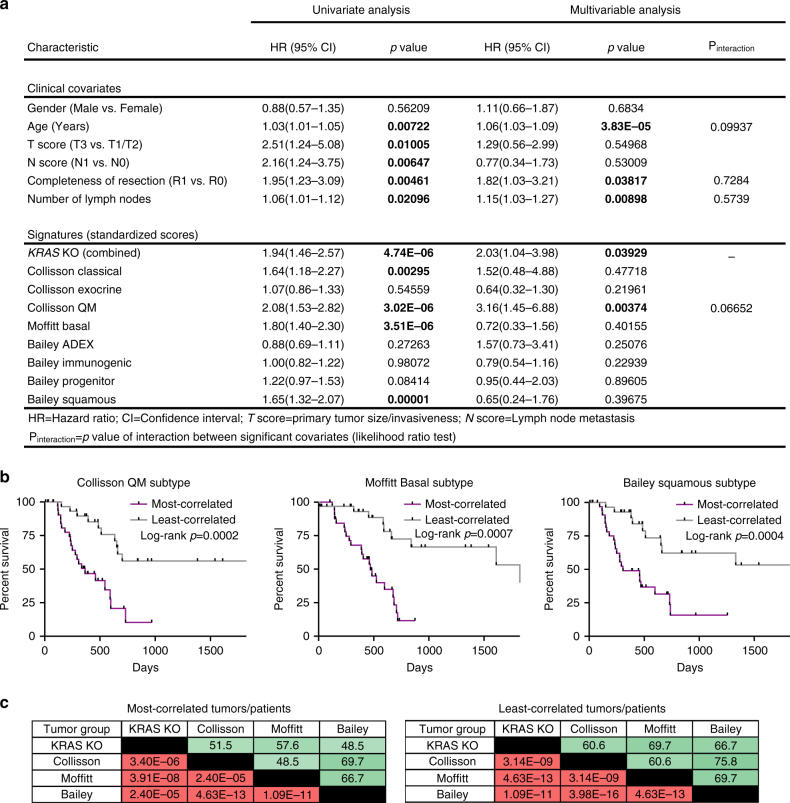
*KRAS* knockout signature overlaps with subtype-specific signatures of PDAC. **a** Univariate and multivariable Cox proportional hazards models on overall survival in the entire TCGA PDAC cohort, including clinical covariates and gene expression signatures, revealed that the combined *KRAS* knockout signature was independently associated with worse survival. Hazard ratios (HR) and *p* values (Cox regression) are reported. A comparison between a model with and without an interaction term (likelihood ratio test) was used to determine independence (*p* > 0.05) between significant covariates in multivariable analysis and the combined *KRAS* knockout signature. **b** Kaplan–Meier plots of survival in human PDAC patients from the TCGA cohort based on tumors most correlated (top quintile) and least correlated (bottom quintile) with the quasi-mesenchymal (QM) subtype from Collisson et al.^21^, basal subtype signature from Moffitt et al.^46^, and the squamous subtype signature from Bailey et al.^45^ (*n* = 33 most correlated and *n* = 33 least correlated tumors). Log-rank (Mantel–Cox) *p* values are shown. **c** Tables show percentage of tumor/patient overlap of top (most correlated) and bottom (least correlated) quintiles of tumors (out of *n* = 33 per group) in each signature. Green shading corresponds to degree of overlap (white = 0%, dark green = 100%). *p* values for significance of overlap (between *n* = 33 groups) within the TCGA PDAC cohort (out of 166 total tumors) are also shown (hypergeometric test). Red shading corresponds to significance of overlap (white = 0.05, dark red = minimum *p* value)

Finally, we examined whether our knockout signature was associated with a particular subtype of pancreatic cancer, as defined by recent gene expression profiling studies^21, 45, 46^. Univariate and multivariable Cox regression analyses suggested that our combined knockout signature and subtype-specific signatures, including the quasi-mesenchymal^21^, basal^46^, and squamous^45^ subtypes defined from three independent human PDAC cohorts, were associated with worse survival within the TCGA PDAC cohort (Fig. 10a). Consistent with these results, Kaplan–Meier survival analysis using our knockout signature mirrored those seen with the subtype-specific signatures (Fig. 10b). Interestingly, we observed statistically significant overlap between the TCGA tumors most correlated (or least correlated) with our knockout signature and tumors most correlated (or least correlated) with these subtype-specific signatures (Fig. 10c). Based on these findings, we hypothesize that the quasi-mesenchymal, basal, and squamous subtypes all define a common set of tumors with distinct biology, impact on survival, and decreased KRAS function.

## Discussion

Here we investigated the consequence of complete endogenous *KRAS* ablation in PDAC cells. By inducing loss-of-function mutations in *KRAS*, we modeled the cellular effects of complete inhibition to predict resistance mechanisms to KRAS inhibition and to uncover KRAS-regulated pathways in PDAC. Given the significant adverse effects of *KRAS* knockout on in vitro proliferation and in vivo tumorigenic growth even in knockout-tolerant cells, our study adds to prior work supporting the merit of KRAS-directed therapies for PDAC^10, 18^. Furthermore, the dose-response relationship between KRAS levels and cellular phenotypes observed (e.g., PANC-1 clones in Fig. 2) highlights the therapeutic benefit of developing the most potent inhibitor possible. With the significant public and private investment toward the generation of novel KRAS-targeted drugs^17, 18^, elucidation of potential resistance mechanisms concurrent with the development of these inhibitors will facilitate their effectiveness in the clinic.

Recent studies have implicated YAP1 overexpression as a means to escape KRAS inhibition in a subset of tumors and cell lines^22, 26^. Our *KRAS* knockout cells revealed an alternative bypass mechanism supported by canonical (i.e., AKT pathway) and non-canonical (i.e., MAPK pathway) PI3K signaling. Given the variety of resistance mechanisms that arise in response to tyrosine-kinase inhibition in RTK-mutated cancers, it is unsurprising that multiple pathways could maintain PDAC cell survival following KRAS inhibition in various contexts. Importantly, we observed PI3K dependence in *KRAS* deficient clones derived from 6/7 (86%) parental cell lines, suggesting that PI3K represents a convergent target regardless of tumor cell line or species of origin.

While PI3K signaling can contribute to PDAC development in mouse models, its role in PDAC maintenance has been less clear. Expression of oncogenic *PIK3CA* in the developing pancreas phenocopies pancreatic cancer initiation and progression observed in *KRAS* mutant mice^47, 48^. In contrast, PI3K inhibition alone has only marginal benefit in preclinical mouse PDAC models, whereas combination therapies with MEK inhibition demonstrate significantly greater anti-tumor effects^49–51^. In accordance, combined MAPK and AKT blockade exhibited by single-agent PI3K inhibitors in PDAC cells is only evident when oncogenic *KRAS* is absent. Although the MAPK pathway dependence on PI3K has been demonstrated previously^31, 32, 52, 53^, we reveal a novel induced dependency on PI3K activity due to loss of oncogenic *KRAS* expression and provide evidence that this rewiring occurs at the level of wild-type RAS.

Despite the fact that PI3K has typically been considered a RAS effector, a growing body of evidence suggests that PI3K can act upstream to stimulate RAS–MAPK signaling in various contexts. Our data demonstrates that *RAS* mutant cancer cells in which mutant *RAS* is ablated or *RAS* WT PDAC cells (e.g., BxPC3) show PI3K-dependent MAPK activation. Recent work from Rosen and colleagues revealed that PI3K inhibition in breast cancer cells harboring oncogenic *PI3K* mutations induces a transient decrease in RAS–MAPK signaling, which could be blunted by expression of exogenous mutant *RAS* ^32^. Interestingly, this response is not limited to cancer cells, as PI3K inhibition in human embryonic kidney cells (Supplementary Fig. 10c) and hematopoietic progenitor cells^54, 55^ also reduced MAPK signaling. Though the precise nature of how PI3K stimulates RAS–MAPK activation in *KRAS* deficient PDAC cells remains unclear, we speculate that this could be due to alterations in GAP activity, recruitment via Gab1/Shp2^56^, or phospholipid signaling^54^. Nonetheless, we show that PI3K transitions to an upstream targetable regulator of not only the canonical AKT pathway, but also the RAS–MAPK pathway when oncogenic *KRAS* is lost. Additionally, we demonstrate that biochemical evidence of transient MAPK inhibition is a better predictive biomarker for PI3K inhibitor sensitivity than PI3K/AKT pathway hyperactivation (Supplementary Figs. 7 and 10b, c).

We further took advantage of *KRAS* intact and deficient cell lines to identify novel KRAS-regulated pathways in PDAC. These include the upregulation of genes associated with ribosomal biogenesis and protein translation and the downregulation of genes associated with interferon response and the metastatic cascade. Moreover, we developed a 16-gene signature, suppressed by KRAS, with independent prognostic value in PDAC validated in two different patient cohorts (TCGA and ICGC). Finally, we demonstrated that our *KRAS* knockout signatures are enriched in PDAC CTCs relative to primary tumors. Given these data and evidence that CTCs display expression of genes downregulated by oncogenic *KRAS* in multiple contexts, we propose that decreased KRAS activity may promote gene expression changes that drive metastasis. Moreover, we hypothesize that the poor prognoses observed with the quasi-mesenchymal, basal, and squamous subtypes is a consequence of decreased KRAS activity leading to greater metastatic potential in these human tumors. Consistent with this hypothesis, epigenetic rather than genetic mechanisms appear to mediate metastasis in human PDAC tumors^57, 58^. While further work is needed in primary human specimens to confirm the relationship between KRAS activity and metastasis, our work highlights KRAS-relevant gene signatures as independent prognostic factors in PDAC.

In summary, we have shown that *KRAS*, the hallmark mutated gene in PDAC, is dispensable in a subset of PDAC cells. We identified novel functions of KRAS in altering gene expression balancing proliferation and metastasis. Finally, our data demonstrate that canonical and non-canonical PI3K pathway activation may bypass the requirement for *KRAS* in PDAC and that simultaneous inhibition of KRAS and PI3K may be a viable combinatorial therapeutic strategy for this disease.

## Methods

### Cell lines and culture conditions

Cell lines used in this study are described in Supplementary Table 2. Established human PDAC cell lines were obtained from the Broad Institute Cancer Cell Line Encyclopedia, sourced from DSMZ-Germany, American Type Culture Collection (ATCC), and RIKEN, and identity authenticated by DNA fingerprinting by the Broad Institute. Primary human PDAC cell lines (PACO9 and PACO19) were obtained as previously described^59^. Briefly, cell lines were derived from pancreatic cancer tissue specimens obtained from patients admitted to the Department of General, Visceral, and Transplantation Surgery at the University of Heidelberg under a protocol approved by the ethical committee of the University of Heidelberg (301/2001) and conducted in accordance with the Helsinki Declaration. Informed consent was obtained from all patients. Mouse PDAC cell lines were derived from primary pancreatic tumors from *LSL-Kras* ^*G12D*^ *; p53* ^*flox/flox*^ *; Pdx1-CreER* mice treated with tamoxifen (Sigma) to induce oncogenic *Kras* ^*G12D*^ activation and biallelic *p53* inactivation in the pancreas^60^ or from the *LSL-Kras* ^*G12D*^ *; p53* ^*R172H/WT*^ *; Pdx1-Cre (KPC)* mouse model^61^. All cell lines tested negative for mycoplasma by PCR testing.

Cells were maintained in DMEM (Corning Cellgro) supplemented with 10% fetal bovine serum (FBS) (Hyclone) and penicillin/streptomycin or RPMI-1640 (Corning Cellgro) supplemented with 10% FBS, L-glutamine, and penicillin/streptomycin. PACO cell lines were grown in serum-free medium as previously described^59^. For inducible-shRNA experiments, doxycycline (DOX, Sigma) was used at 1 μg mL^−1^ in culture media and replaced every 2–3 days. Cell viability was analyzed after 5 days of DOX treatment using the CellTiter-Glo (CTG) luminescence assay (Promega), which measures cellular ATP levels as a surrogate for cell number and growth. Luminescence was read on a Tecan M2000 Infinite Pro plate reader. Cells were imaged with a Nikon Eclipse TE2000-U light microscope and SPOT RT3 camera.

### Cloning of sgRNA and overexpression constructs

Lentiviral constructs for CRISPR/Cas-mediated genome editing from the dual-vector lentiviral GeCKOv2 system, *lentiCas9-Blast* and *lentiGuide-Puro* ^62^ (Supplementary Fig. 1a), were provided by Dr Feng Zhang. sgRNAs targeting human and mouse *KRAS* exons were designed (Supplementary Table 1) and ligated into the BsmBI site with compatible annealed oligos. *lentiGuide-Hygro* (Supplementary Fig. 1a) was generated following sgRNA ligation by subcloning a hygromycin resistance gene PCR amplified from *MSCV-Luciferase-pPGK-Hygro* (Addgene #18782) into BsiWI and MluI sites. pLKO-Tet-On constructs (Supplementary Fig. 1c) targeting KRAS (shKRAS.407) and LACZ (shLACZ.1650) and pLKO constructs targeting YAP1 (shYAP1.1573 and shYAP1.1928) and LACZ (shLACZ.1650) were provided by Dr William Hahn (Supplementary Table 1). *mmKras*.*366* sgRNA targeting mouse *Kras* was cloned into a lentiviral vector for DOX-inducible sgRNA expression provided by Dr Marco Herold^63^. To generate lentiviral constructs for overexpression, we first produced *LV-pSV40-mCherry-pPGK-FlpO*, *LV-pSV40-Puro-pPGK-eGFP*, and *LV-pSV40-Blast-pPGK-eGFP* by assembling four parts with overlapping DNA ends into a 5.7 kb lentiviral backbone using Gibson assembly (New England Biolabs) as previously described^64^. PCR amplified cDNAs (Supplementary Table 5) were subcloned into AfeI and AscI sites to replace *FlpO* and *eGFP* and verified by sequencing and restriction digest. Alternatively, cDNAs were subcloned into the EcoRI and BamHI sites of *pBABE-zeo* (Addgene #1766) or *pBABE-puro* (Addgene #1764) retroviral vectors. The translation reporter construct *MSCV-mCherry-IRES-GFP* was generated by subcloning PCR amplified mCherry into the XhoI and EcoRI sites of *MSCV-IRES-GFP* (Addgene #9044). High-fidelity restriction enzymes (New England Biolabs) were used for restriction digests when available. Cloning methods and primer sequences not detailed in this manuscript are available upon request.

### Lentiviral and retroviral production and transduction

For lentiviral infections, lentiviral backbone, packaging vector (delta8.2 or psPAX2), and envelope (VSV-G) were transfected into 293T cells with TransIT-LT1 (Mirus Bio). Supernatant was collected at 48 and 72 h and applied to target cells with 8 μg mL^−1^ polybrene (EMD Millipore) for transduction. Transduced cells were treated with 10 μg mL^−1^ blasticidin S (Life Technologies), 2–4 μg mL^−1^ puromycin (Life Technologies), 400 μg mL^−1^ hygromycin B (Roche), or 400 μg mL^−1^ zeocin (Life Technologies) for 3–7 days, as appropriate, for antibiotic selection. Alternatively, *Cas9-Blast*-expressing cells were transfected with *lentiGuide-Puro* harboring *sgKRAS* to induce *KRAS* knockout (8988T T1 and T2 cells) using the Amaxa Nucleofector Kit for Mammalian Epithelial Cells (Lonza). To generate single cell clones from *sgKRAS*-transduced cells, we sorted one cell per well into 96-well plates using a FACSAria II (Becton Dickinson) or MoFlo (Beckman Coulter) FACS sorter. Alternatively, PACO clones were manually picked from culture plates following low-density plating. Intact clones were derived from *lentiGuide-Puro-* or *lentiGuide-Hygro*-transduced cells without inserted guide sequence (empty vector) except for 8988T E1, which was derived from *hsKRAS*.*22V*-transduced cells but exhibited loss of Cas9 protein expression, no mutagenesis, and *KRAS* intact cell properties. *sgKRAS* vectors used to generate knockout clones are listed in Supplementary Table 3. For overexpression constructs harboring *mCherry*, fluorescent cells were sorted using a FACSAria II sorter. For retroviral infections, the retroviral backbone and pCL-Eco (for mouse cells) or pUVMC and VSV-G (for human cells) were transfected into 293T cells. For the translational reporter experiments, fluorescence was assessed using an LSR II FACS analyzer (Becton Dickinson) and data were analyzed using FlowJo software.

### In vitro growth assays

Anchorage-independent growth was assessed by plating 10,000 cells in 0.4% low melting temperature agarose (Seaplaque) in complete media on top of a 0.8% preformed agarose layer. Cells were grown for 9–14 days and colonies were stained with 0.5% crystal violet and destained with water. Microscopic images of the colonies were taken pre- and post-crystal violet staining. For growth curves, 250–1000 cells were plated on day 0 and grown for 5 days in culture. Four–five replicates for each cell line per day were assessed for cell viability by CTG assay. Cell viability results were normalized to luminescence at day 0. Three-dimensional culture was established by plating 250–500 cells from a single cell suspension onto a growth factor-reduced matrigel (Corning) layer, allowing cell migration into matrigel for 4–6 h. Cells were grown in complete media for 7–10 days prior to analysis. Three technical replicates were performed for each clone. Apoptosis was measured using the Guava Nexin Reagent per the manufacturer’s instructions and analyzed on a Guava flow cytometry system (Millipore).

### Immunoblotting

Cells were lysed with ice-cold RIPA buffer (Pierce), supplemented with 0.5 μM EDTA and Halt protease and phosphatase inhibitors (Thermo Scientific), rotated at 4 °C for 15–30 min to mix, and centrifuged at maximum speed for 15 min to collect whole cell lysates. Protein concentration was measured with the BCA protein assay (Pierce). An aliquot of 20–30 μg of total protein per sample was loaded into 4–12% Bis–Tris gradient gels (Life Technologies) and separated by SDS-PAGE. Proteins were transferred to nitrocellulose (for LI-COR) or PVDF (for ECL) membranes. The following antibodies were used for immunoblotting: mouse anti-HSP90 (BD #610418, 1:10,000), rabbit anti-beta-tubulin (CST 2128, 1:1000), mouse anti-KRAS (SCBT sc-30, 1:200), mouse anti-NRAS (SCBT sc-31, 1:200), mouse anti-HRAS (SCBT sc-29, 1:200), rabbit anti-pERK1/2(T202/Y204) (CST 4370, 1:1000), mouse anti-ERK1/2 (CST 9107, 1:1000), rabbit anti-pAKT(S473) (CST 4060, 1:2000), rabbit anti-pAKT(T308) (CST 2965, 1:1000), mouse anti-AKT (CST 2966, 1:2000), rabbit anti-pPRAS40(T246) (CST 2997, 1:1000), rabbit anti-PTEN (CST 9559, 1:1000), rabbit anti-INPP4B (Abcam ab81269, 1:1000), rabbit anti-pS6(S235/236) (CST 4858, 1:2000), mouse anti-S6 (CST 2317, 1:1000), rabbit anti-4EBP1(S65) (CST 9451, 1:1000), rabbit anti-pCRAF(S338) (CST 9427, 1:1000), rabbit anti-pMEK1/2(S217/221) (CST 9154, 1:1000), mouse anti-MEK1/2 (CST 4694, 1:1000), rabbit-anti-c-MYC (Abcam ab32072, 1:1000), rabbit anti-c-MYC (CST 5605, 1:1000), and rabbit anti-YAP1 (CST 4912, 1:1000). HSP90 and beta-Tubulin were used as loading controls. RAS-GTP assays were performed using an Active RAS Pull-Down and Detection Kit (Thermo Scientific 16117) per the manufacturer’s instructions using in vitro GTPγS (non-hydrolysable) and GDP pull-down controls and the provided pan-RAS antibody (1:200). Mouse RTK arrays (R&D Systems) were assayed per the manufacturer’s instructions. Primary antibodies were detected with fluorescent DyLight-conjugated (CST) or HRP-conjugated (BioRad) secondary antibodies for fluorescent (LI-COR) or chemiluminescent detection (Amersham), respectively. Approximate locations of protein size markers (in kDa) are shown in individual blots. Quantification of protein levels from western blots was performed using Image Studio Lite (LI-COR).

### Genomic DNA isolation and sequencing

Genomic DNA from cell lines was collected using QuickExtract DNA extraction solution (Epicentre) or a QiaAMP DNA Mini Kit (Qiagen). PCR products for sequencing were amplified using a Herculase II Fusion DNA polymerase kit (Agilent) and primers described in Supplementary Table 6. PCR products from cell lines were subject to Sanger sequencing (Quintara Biosciences). For subclones with multiple *KRAS* mutant alleles, PCR products were cloned into a TOPO vector (Life Technologies) and at least 10–20 bacterial colonies were sequenced per cell line. The most probable off-target genes for sgRNAs were identified using CRISPR Design (http://crispr.mit.edu) and no gene had fewer than three exonic mismatches. All sequences and chromatograms were analyzed using MacVector software.

Genomic DNA was isolated from ground flash frozen tumor tissue using a High Pure PCR Template Preparation Kit (Roche). Mouse *Kras* exon 3 was PCR amplified using Herculase II Fusion DNA polymerase. Sequencing libraries were prepared from 50 ng of PCR product using the Nextera DNA Sample Preparation Kit (Illumina) and sequenced with Illumina MiSeq. Sequencing reads (150 bp paired-end) were trimmed to 120 bp after reviewing base quality profiles, in order to drop lower quality 3′ ends. Traces of Nextera adapters were clipped using the FASTX toolkit (Hannon Lab, CSHL) and pairs with each read greater than 15 bp in length were retained. Additionally, read pairs where either read had 50% or more bases below a base quality threshold of Q30 (Sanger) were dropped from subsequent analyses. The reference sequence of the target locus was supplemented with 10 bp genomic flanks and was indexed using an enhanced suffix array. Read ends were anchored in the reference sequence using 10 bp terminal segments for a suffix array index lookup to search for exact matches. A sliding window of unit step size and a maximal soft-clip limit of 10 bp was used to search for possible anchors at either end of each read. For each read, optimal Smith-Waterman dynamic programming alignment was performed between the reduced state space of the read sequence and the corresponding reference sequence spanning the maximally distanced anchor locations. Scoring parameters were selected to allow for sensitive detection of short and long insertions and deletions while allowing for up to four mismatches, and the highest scoring alignment was selected. Read pairs with both reads aligned in the proper orientation were processed to summarize the number of wild-type reads and the location and size of each insertion and deletion event. Overlapping reads within pairs were both required to support the event if they overlapped across the event location. Additionally, mutation events and wild-type reads were summarized within the extents of the sgRNA sequence and PAM site by considering read alignments that had a minimum of 20 bp overlap with this region. Mutation calls were translated to genomic coordinates and subsequently annotated using Annovar (http://annovar.openbioinformatics.org). The alignment and post-processing code was implemented in C++ along with library functions from SeqAn (https://www.seqan.de/) and SSW^65^ and utility functions in Perl and in the R statistical programming language (www.R-project.org). Mutation calls were subjected to manual review using the Integrated Genomics Viewer (IGV; http://software.broadinstitute.org/software/igv/).

### Quantitative PCR for copy number analysis

Quantitative PCR of the *KRAS* locus and control regions (*LINE1* for human, *chromosome 5 10054507–10054621* region for mouse) was performed as previously described^66, 67^ in triplicate using SYBR green reagents (Life Technologies) with primers listed in Supplementary Table 7. Genomic DNA from 293T and murine ES cells were used as control cells to determine 2N copy number for human and mouse samples, respectively. *C*_p_ values were measured by a LightCycler 480 Real-Time PCR System (Roche). *KRAS* DNA relative to control region (*KRAS* ^*amp*^) was calculated for samples and control cell lines using the following formula: 2^(*KRAS* Cp-Control region Cp)^. *KRAS* copy number was determined using the following formulation: 2 x *KRAS* ^*amp*^ (sample)/*KRAS* ^*amp*^ (control cell line).

### High-throughput drug screening

High-throughput screening was performed as previously described^68^. To determine the optimal plating density during assay development, 8988T E3, E6, H9, and H36 cells were plated at either 500, 1000, or 1500 cells per well into 384-well opaque, white assay plates (Corning), 50 μL per well, and incubated overnight at 37 °C/5% CO_2_. The next day, cells were treated with MG-132 (Enzo Bioscience) starting at a high concentration of 40 μM, in a 14-pt, twofold dilution series, 16 replicates/concentration, for 72 h. 0.1% DMSO (solvent for all compounds) was used as a negative control. Sensitivity was assayed using CTG. Luminescence was measured using a M1000 Infinite Pro plate reader (Tecan). The Z′ factor at each concentration point was calculated and compared between each cellular density to determine the largest dynamic detection window for subsequent screening. Estimated Z′ factors were calculated using the following formula: 1 − (3 × (*σ*_p_ + *σ*_n_)/(*μ*_p_ − *μ*_n_)) where *σ* (standard deviation) and *μ* (mean) were determined from the positive (p) and negative (n) controls. For the screen, cells were plated at a density optimized during assay development as above. A modified version of the Selleck Cambridge Cancer Compound Library (http://www.selleckchem.com/screening/cambridge-cancer-compound-library.html) containing 384 structurally diverse, medicinally active, and cell permeable cancer-relevant compounds was used for screening. Compounds were plated in 384-well format in 5-pt, 10-fold concentration ranges, starting at 10 mM. 50 nL of compounds were pin-transferred (V&P Scientific pin tool mounted onto a Tecan Freedom Evo 150 MCA96 head) into duplicate assay plates and incubated for 72 h. The DMSO content was 0.1% within each well. Thirty-two wells of DMSO vehicle control and 32 wells of positive control MG-132 were included on each plate. After 3 days of incubation, 10 μL of CTG was added to each well, incubated for 10 min, and luminescence output was read as a surrogate for cell viability. Z′ factors were >0.5 for all plates in the screen. Percent viability (PV) compared to DMSO control was calculated for each compound well and plotted against log_10_(Dose) (M). Area under the curve (AUC) was calculated using the trapezoidal rule: ((PV_1_ + PV_2_)/2) × (dose_1_ − dose_2_). AUCs were averaged for intact (AUC_i_) and knockout (AUC_KO_) cells across replicates. Compounds were considered hits if (1) AUC_i_ or AUC_KO_ were <4; (2) ΔAUC (AUC_i_ – AUC_KO_) was >0.5 or <−0.5; and (3) AUC_i_ and AUC_KO_ were significantly different (*p* < 0.05, Student’s *t* test).

### Drug treatments

GDC-0941, BAY80-6946, AZD6244, MK2206, AZD8055, BYL719, TGX220, Idelalisib, BGJ-398, and Crenolanib were purchased from Selleck Chemical. Verteporfin was purchased from Sigma. All compounds were diluted to 10–50 mM stock concentration in DMSO except for BAY80-6946, which was diluted to 5 mM in DMSO with 10 mM trifluoroacetic acid (TFA). To generate dose-response curves, cells (500–1000 for A13 clones, 2000–4000 for PANC-1 clones and primary hPDAC clones, and 1000–2000 for 8988T, KP-4, and MM1402 clones) were plated in 96-well white plates (Perkin Elmer) in 100 μL of media and incubated overnight. Hundred microliter of drug at 2× final concentration was added to each well for 3–6 replicates for each cell line and dose. Cell viability was determined at 72 h using CTG. For ligand treatments, cells were treated with recombinant EGF, FGF1, or PDGF-BB (R&D Systems) when initially plated. Percent viability was calculated for each dosed well compared to solvent controls (DMSO or DMSO with TFA at 0.1–0.2%) and plotted against log_10_(Dose) (M). For dose-response curves, each replicate for each cell line and dose was plotted along with curve fit regression for three-component inhibitor response (Prism). For long-term drug treatments, cells were plated at low-density in six-well plates in triplicate, treated with drugs for 10–14 days (media was refreshed with drug every 2 days), and stained with 0.5% crystal violet when control cells became confluent.

### Animal studies

All animal studies were approved by the MIT Institutional Animal Care and Use Committee. A13 mouse clones were transplanted to form tumors in nude mice (Taconic) via subcutaneous injections. A total of 5 × 10^5^ cells suspended in 100 μL of cold PBS were injected per tumor to determine tumor-forming capacity. For 8988T and PANC-1 clones, 2 × 10^6^ cells suspended in 100 μL of cold 3:1 PBS:growth factor-reduced matrigel (Corning) were injected per tumor. For drug treatment of A13 tumors in vivo, a higher number of knockout cells (2 × 10^6^) was injected to synchronize tumor formation between intact and knockout clones, as 5 × 10^5^ cells of K1 and K2 give rise to subcutaneous tumors but at significantly slower rates than E1 and E2. Tumor formation was monitored over time by direct observation, caliper measurement, and IVIS spectrum optical imaging (Xenogen Corporation). When subcutaneous tumors grew to 0.5 cm in diameter (approximate bioluminescent radiance of 1 × 10^10^ photons s^−1^ cm^−2^ per sr), mice were randomly dosed (based on ear tag number assigned at time of tumor transplant) with 150 mg kg^−1^ of GDC-0941 (LC Laboratories) or vehicle alone (10% DMSO and 5% Tween-20 in nuclease-free water) daily for 14 days by oral gavage. We estimated that to observe a 50% reduction in tumor growth at 2 weeks after initiation of treatment (mean normalized volume ± s.d. of 12 ± 4) with alpha of 0.05 and power of 0.8, we would need at least seven tumors per group.

For transplant of A13 cells harboring DOX-inducible *mmKras*.*366*, 5 × 10^5^ cells suspended in 100 μL of cold PBS were injected per tumor subcutaneously. Clone 1 was injected to the left flank and clone 2 to the right flank of each immunocompromised mouse. DOX feed (Harlan-Teklad) was administered after all the tumors were 0.5 cm in diameter as monitored by caliper measurements. After 4 days of DOX feed, 150 mg kg^−1^ of GDC-0941 or vehicle alone was dosed daily for 14 days by oral gavage. Caliper measurements and bioluminescence imaging by IVIS was done in 3–4 day intervals by injecting 100 μl of 30 mg ml^−1^ luciferin per mouse and imaging 10 min post injection. The level of bioluminescence in radiance was analyzed by Living Image software (Perkin Elmer). Tumor volume was calculated from caliper measurements using the modified ellipsoid formula: (length) × (width)^2^/2. Investigators were not blinded to treatment groups during protocol. Bliss independence for additive effect of DOX-induced *KRAS* knockout and GDC-0941 treatment was calculated for each measured time point using the following formulas: Fractional response to GDC-0941 (F_GDC_) = (1−(TV_GDC_/TV_Veh_)), Fractional response to DOX-induced *KRAS* knockout (F_DOX_) = (1−(TV_DOX_/TV_Veh_)), additive fractional response to GDC-0941 and DOX (F_GDC+DOX_) = F_GDC_ + F_DOX_ − (F_GDC_ × F_DOX_). Bliss line corresponds to tumor volume (TV) with predictive additive effect of treatments and determined with the following equation at each time point: TV_Veh_(1 − F_GDC + DOX_).

### RNA isolation and sequencing analysis

RNA was isolated from PDAC cells using TRIzol (Life Technologies). cDNA libraries were prepared using an Illumina TruSeq sample preparation kit with indexed adapter sequences and polyA selection. Sequencing was performed on an Illumina HiSeq 2000 instrument to obtain single-end 50-nt reads. All reads that passed quality metrics were mapped to the UCSC mm9 mouse or hg19 human genome build (http://genome.ucsc.edu/) using RSEM (v1.2.12) (http://deweylab.github.io/RSEM/). For pairwise differential expression analyses, data normalization (MedianNorm) and differential analyses between experimental conditions were performed using EBSeq v1.4.0 (http://bioconductor.org/packages/release/bioc/html/EBSeq.html). All RNA-Seq analyses were conducted in R. Unsupervised clustering was performed using a Pearson correlation-based pairwise distance measure. Heat maps were generated using the Heatplus package in R.

High-resolution signature analyses between clones within each cell line were performed using a blind source separation methodology based on ICA^69^. RSEM generated estimated expression counts were upper quartile normalized to a count of 1000. The R implementation of the core JADE algorithm (Joint Approximate Diagonalization of Eigenmatrices)^70^ was used along with custom R utilities. Signatures were visualized using the sample-to-signature correspondence schematic afforded by Hinton plots, where colors represent relative directionality of gene expression (red relatively upregulated, blue relatively downregulated within each signature) and the size of each rectangle quantifies the strength of a signature (column) in a given sample (row). Each signature is two-sided, allowing for identification of upregulated and downregulated genes for each signature within each sample. Biologically relevant and statistically significant signatures were identified using a Mann–Whitney *U*-test. Signature correlation scores (*z*-scores) for each gene in the statistically significant signatures are included as supplementary tables. Heat maps were plotted with the top and bottom 2% genes in each signature. Additionally, genes with standardized signature correlation scores *z* > 3 (alternatively *z* < −3) were used as gene sets to score TCGA (https://tcga-data.nci.nih.gov/tcga/) Pancreatic adenocarcinoma (PAAD) tumors using ssGSEA. Tumors were stratified based on standardized signature scores (*z*-scores) derived using ssGSEA and associated patients within matching top and bottom percentile buckets were compared for a statistically significant difference in survival times. Significance of overlap between tumor buckets scored using 8988T, A13, combined knockout, quasi-mesenchymal^21^, basal^46^, and squamous^45^ subtype signatures was assessed using the hypergeometric test. Survival analyses were conducted using the survival package in R.

In order to derive a combined knockout signature, the top and bottom 2% genes in the human signature were analyzed in the mouse expression data set and ICA analysis revealed a WT/KO signature. The up and down gene sets were determined using a standardized signature correlation score of *z* > 0.5 (alternatively, *z* < −0.5). These were used in survival analyses similar to those described above for TCGA and also for ICGC Pancreatic Cancer Australia (PACA-AU) tumors (https://icgc.org/icgc/cgp/68/304/798).

Publicly available microarray data sets were used to generate KRAS-ON and KRAS-OFF signatures from a mouse model of DOX-regulated *KRAS* transgene expression^22, 39, 40^. Array CEL files were retrieved from GEO (GSE32277, GSE53169, and GSE58307) and processed using Affymetrix Power Tools v. 1.15.0 (rma-sketch). Probes were collapsed (max. value) to yield per gene expression estimates. Genes with upper quartile log_2_ expression value less than 5 across all samples were dropped from further analysis. The resulting data sets were used for signature analysis with ICA.

Publicly available RNA-Seq data sets were used to generate the CTC signature^44^. Read counts for the sample set (number of raw reads mapped per gene) were downloaded from GEO for record GSE51372. Entries with duplicate symbols or missing gene names were dropped from further consideration. Samples with less than 5 million total mapped reads were dropped from the data set in order to eliminate expression noise from low coverage. Only samples identified as tumor or classical CTC were retained for downstream analyses. Read counts for the remaining samples were normalized using quartile normalization with the upper quartile set to 1000. In the resulting expression data set, genes with an upper quartile of expression count less than 1000 across all samples were tagged as lowly expressed genes and dropped. Normalized expression values were log_2_ transformed and used as input for signature analysis using ICA.

GSEA were carried out using the pre-ranked mode using log_2_ fold-change values (for pairwise analyses) or standardized signature correlation scores (for ICA signatures) with default settings^38^. Network representations of GSEA results were generated using EnrichmentMap (http://www.baderlab.org/Software/EnrichmentMap) for Cytoscape v3.3.0 (http://www.cytoscape.org) with *p* value and FDR cutoffs as described in figure legends. Each circle represents a gene set with circle size corresponding to gene set size and intensity corresponding to enrichment significance. Red is upregulated and blue is downregulated. Each line corresponds to minimum 50% mutual overlap with line thickness corresponding to degree of overlap. Cellular processes for gene set clusters were manually curated.

Candidate point mutations in RNA-Seq data sets were called using a pipeline based on the GATK Toolkit (https://software.broadinstitute.org/gatk/). Transcriptomic reads were mapped (to mm9, hg19) using the Tophat (v2.0.4) spliced aligner and subjected to local realignment and score recalibration using the GATK Toolkit. Mutations were called in KO samples (individual and pooled) against WT samples (individual and pooled) with a minimum base quality threshold of 30. Genomic annotations were performed using ANNOVAR (http://www.openbioinformatics.org/annovar/).

### Statistical analyses

*P* values for comparisons of two groups were determined by two-tailed Student’s *t* test (for normally distributed data) or Mann–Whitney *U*-test (for non-parametric data) as noted in the figure legends. All replicates were included in these analyses. The log-rank test was used for Kaplan–Meier survival analyses. The Cox regression model (in univariate and multivariable settings) was used to estimate hazard ratios in survival analyses. A *p* value of < 0.05 was used to denote statistical significance. All error bars denote standard error of mean (s.e.m.) or standard deviation (s.d.) as noted in the figure legends.

### Code availability

Computer code for RNA-Seq independent component analyses is available upon request. Other software tools for RNA-Seq analyses, website source, and version numbers are listed above.

### Data availability

The RNA and DNA sequencing data sets that support the findings of this study have been deposited in the Gene Expression Omnibus (GEO) and the NCBI Sequencing Read Archive (SRA) under accession GSE71876. Previously published data sets used in this study are available in GEO under accession GSE32277, GSE53169, GSE58307, and GSE51372. The authors declare that all other data are available within the article and its supplementary information files or available from the corresponding author upon request.

## Electronic supplementary material

Supplementary Information

Peer Review File

Supplementary Data 1

Supplementary Data 2

Supplementary Data 3

Supplementary Data 4

Supplementary Data 5

Supplementary Data 6

Supplementary Data 7

Supplementary Data 8

Supplementary Data 9

Supplementary Data 10

Supplementary Data 11

Supplementary Data 12

Supplementary Data 13

Supplementary Data 14

Supplementary Data 15

Supplementary Data 16

Supplementary Data 17

Supplementary Data 18
