# Supplementary material for: Survival of pancreatic cancer cells lacking KRAS function
Source: Nat Commun. 2017 Oct 23;8:1090. doi: 10.1038/s41467-017-00942-5 (PMC5653666; doi:10.1038/s41467-017-00942-5)
Supplement: Supplementary file 1 — Supplementary Information [file 41467_2017_942_MOESM1_ESM.pdf]

Description of Supplementary Files

File Name: Supplementary Information
Description: Supplementary Figures, Supplementary Tables and Supplementary References

File Name: Supplementary Data 1
Description: RNA-Seq pooled SNP analysis of 8988T KRAS intact and knockout clones

File Name: Supplementary Data 2
Description: RNA-Seq pooled SNP analysis of A13 Kras intact and knockout clones

File Name: Supplementary Data 3
Description: Modified Selleck Cambridge Chemical Library Compound Set used in drug screen

File Name: Supplementary Data 4
Description: AUC response to drugs in screen. AUC for each cell line and replicate, average AUC across intact and knockout clones, AUC difference between intact and knockout clones, and p-values from two-tailed student's t-test are reported

File Name: Supplementary Data 5
Description: Raw and normalized RNA-Seq expression for 8988T KRAS intact and deficient clones. Estimated expression counts (from RSEM), normalized values (from EBSeq), and differential expression results (FDR, fold-change, log2 fold-change, and direction of fold-change) are shown.

File Name: Supplementary Data 6
Description: Raw and normalized RNA-Seq expression for A13 Kras intact and deficient clones. Estimated expression counts (from RSEM), normalized values (from EBSeq), and differential expression results (FDR, fold-change, log2 fold-change, and direction of fold-change) are reported.

File Name: Supplementary Data 7
Description: ICA analysis results for 8988T clones. Normalized log2FC expression values and Z-scores for IC1 signature are shown.

File Name: Supplementary Data 8
Description: ICA analysis results for A13 clones. Normalized log2FC expression values and Z-scores for IC1 signature are reported.

File Name: Supplementary Data 9
Description: Oncogenic signature gene set enrichment analysis (MSigDB C6) of 8988T KRAS deficient cells. Gene sets associated with expression of oncogenic KRAS or MEK in primary epithelial cells demonstrated anti-correlation with the knockout signature (bold).

File Name: Supplementary Data 10
Description: Oncogenic signature gene set enrichment analysis (MSigDB C6) of A13 Kras deficient cells

File Name: Supplementary Data 11
Description: Oncogenic signature gene set enrichment analysis (MSigDB C6) of 8988T KRAS intact cells. Gene sets associated with expression of oncogenic KRAS, MYC, or EIF4E demonstrated correlation with the intact signature.

File Name: Supplementary Data 12
Description: Oncogenic signature gene set enrichment analysis (MSigDB C6) of A13 Kras intact cells. Gene sets associated with expression of oncogenic KRAS in primary epithelial cells demonstrated correlation with the intact signature.

File Name: Supplementary Data 13
Description: ICA analysis derived from DOX-inducible Kras transgenic model studies. IC4 signature derived from Ying et al[1] was associated with acute KRAS transgene withdrawal. IC8 signature derived from Viale et al[2] was associated with surviving cells following KRAS transgene withdrawal. IC6 signature derived from Kapoor et al.[3] was associated with relapsed KRAS-independent tumors following long-term KRAS transgene withdrawal. Z-scores for each ICA signature are shown.

File Name: Supplementary Data 14
Description: Curated gene set enrichment analysis (MSigDB C2) of 8988T KRAS deficient cells

File Name: Supplementary Data 15
Description: Curated gene set enrichment analysis (MSigDB C2) of 8988T KRAS intact cells

File Name: Supplementary Data 16
Description: Curated gene set enrichment analysis (MSigDB C2) of A13 Kras deficient cells

File Name: Supplementary Data 17
Description: Curated gene set enrichment analysis (MSigDB C2) of A13 Kras intact cells

File Name: Supplementary Data 18
Description: TCGA survival analysis

File Name: Peer Review File

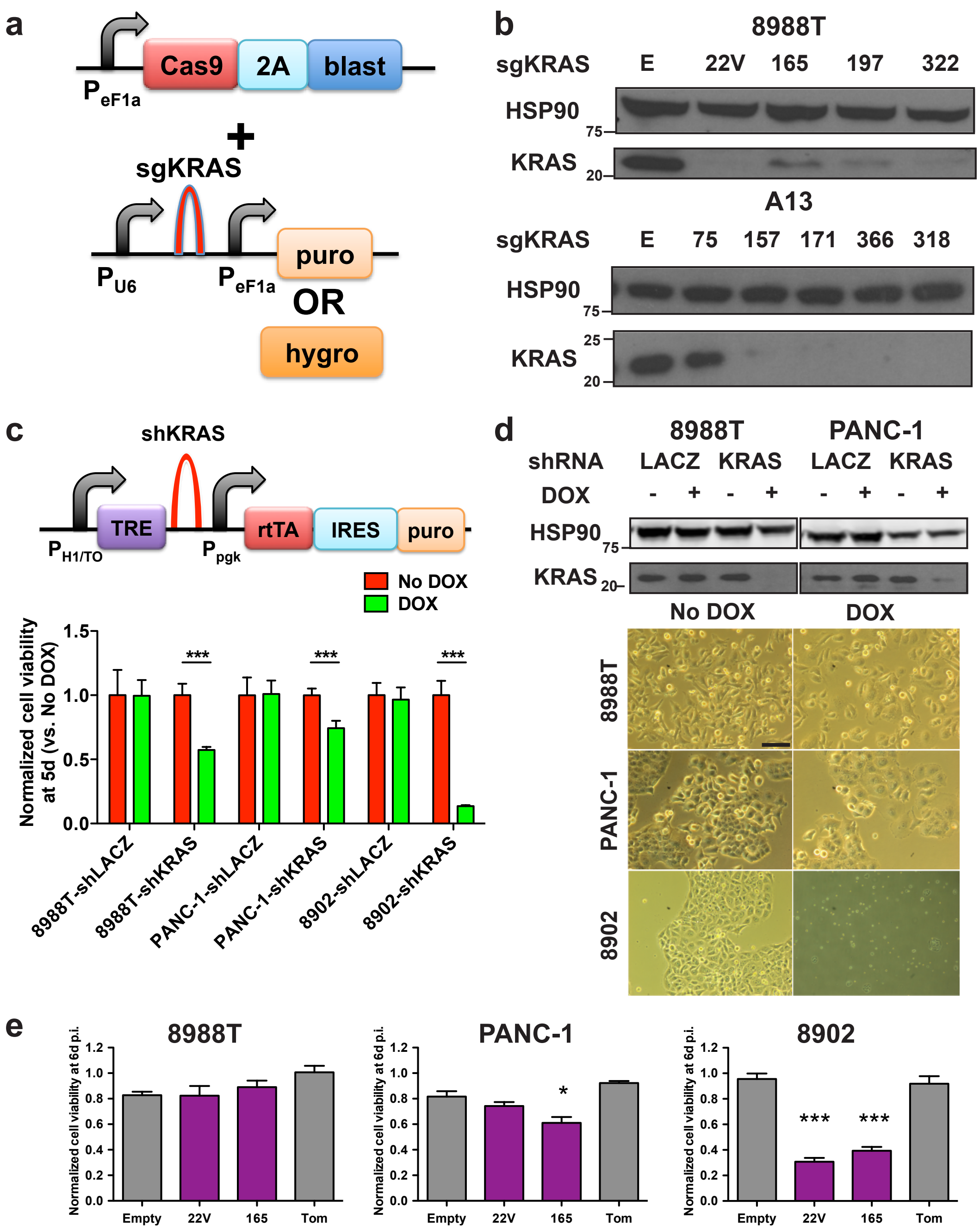

Supplementary Fig. 1. ***KRAS*** **knockdown and knockout in PDAC cells.**

a) Schematic of lentiviral constructs used to express spCas9 nuclease and sgRNAs in PDAC cells. P_{eF1a} = ubiquitously expressed elongation factor 1a promoter. P_{U6} = RNA polymerase III human U6 promoter. 2A = self-cleaving peptide. Blast = blasticidin resistance gene. Puro = puromycin resistance gene. Hygro = hygromycin resistance gene.

b) Western blot of KRAS levels assayed 7 days after transduction of a population of spCas9-expressing 8988T and A13 cells with sgRNAs targeting *KRAS* (sgKRAS). Residual protein is likely due to inefficient cutting or in-frame mutagenesis in some cells within the population. See **Supplementary Table 1** for sgKRAS sequences.

c) Schematic of doxycycline (DOX)-inducible shRNA lentiviral construct pLKO-Tet-On. TRE = tetracycline-responsive element. P_{pgk} = ubiquitously expressed phosphoglycerate kinase promoter. rtTA = reverse tetracycline transactivator. IRES = internal ribosomal entry site. 8988T and PANC-1 cell lines exhibited a modest but significant decrease in cell viability after 5 days of *shKRAS.407* induction with DOX, while 8902 cells showed a marked decrease in viability. Average cell viability (normalized to no DOX condition) +/- s.d. (n=5 replicates per condition) is plotted. ***p<0.001, two-tailed Student's t-test comparing *shKRAS.407* to *shLACZ.1650*. See **Supplementary Table 1** for shRNA sequences.

d) Western blot of KRAS protein levels following 7 days of DOX treatment with 8988T and PANC-1 cell lines transduced with *shLACZ.1650* or *shKRAS.407*. KRAS knockdown induced alterations in cell morphology in 8988T and PANC-1 cells and apoptosis in 8902 cells. Scale bar is 100 µm.

e) Cell viability 6 days following sgRNA transduction with empty vector, *hsKRAS.22V*, *hsKRAS.165*, or *sgTomato* control vector. Cells were maintained in puromycin following transduction to ensure sgRNA transduction. Average cell viability (normalized to unselected cells) +/- s.d. (n=4 replicates per condition) is plotted. *p<0.05, ***p<0.001, two-tailed Student's t-test comparing sgRNA with empty vector control.

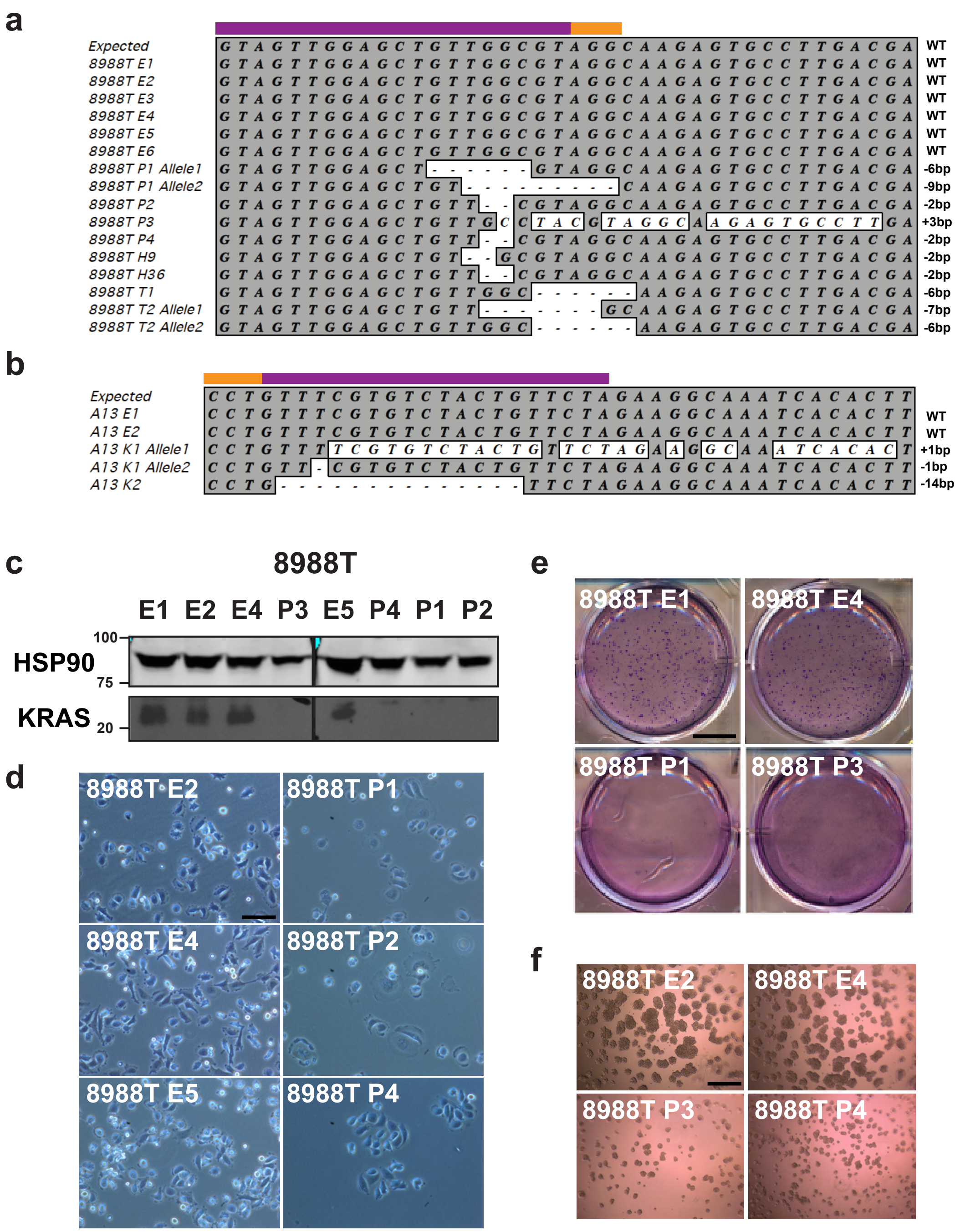

Supplementary Fig. 2. Characterization of 8988T *KRAS* deficient clones.

a) *KRAS* alleles from 8988T clones showed indels in knockout clones. Expected sequence corresponds to reference from UCSC hg19 and was observed in all intact clones (E1-E6). All knockout clones retained a single indel allele except for P1 and T2 for which two different indels were identified. Both out-of-frame (P2, P4, H9, H36, T2 allele 1) and in-frame indels

compromising essential codons 12 or 13 (P1, P3, T1, and T2 allele 2) were observed in sequenced knockout clones. The purple and orange bars denote the sgRNA and PAM sequences, respectively.

b) *KRAS* alleles from A13 clones showed indels in knockout clones. Expected sequence corresponds to reference from UCSC mm9 and was observed in intact clones (E1, E2). K1 carried a heterozygous 1bp insertion and deletion, while K2 harbored a single 14bp out-of-frame deletion.

c) Western blot showed loss of KRAS protein in 8988T knockout clones (P1, P2, P3, P4) compared to intact clones (E1, E2, E4, E5).

d) 8988T *KRAS* deficient clones exhibited altered cell morphology, characterized by increased cell size, cytoplasmic translucency, and smooth edges. Scale bar is 100 µm.

e) 8988T *KRAS* deficient clones showed decreased anchorage-independent growth in soft agar. Scale bar is 1 cm.

f) 8988T *KRAS* deficient were capable of growing in 3D culture in matrigel but exhibited smaller sphere size compared to intact clones 7 days after single cell suspension plating, consistent with decreased proliferation. Scale bar is 500 µm.

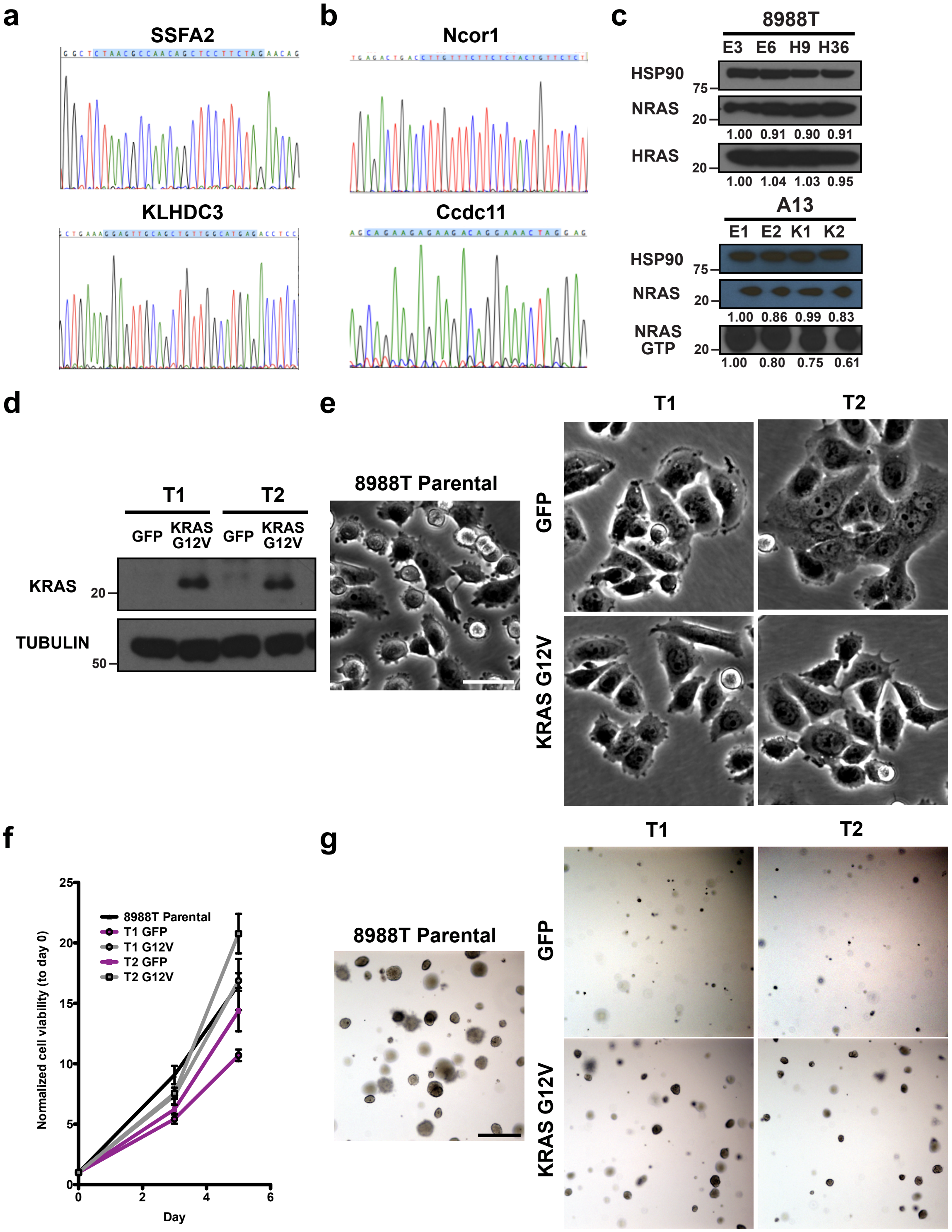

Supplementary Fig. 3. On-target effects of sgRNAs targeting *KRAS*.

a) Representative Sanger sequencing results of the top two exonic mismatch genes for human sgRNA *hsKRAS.22V* revealed no mutational changes in *KRAS* knockout clones. Potential off-target sequences are highlighted in blue.

b) Representative Sanger sequencing results of the top two exonic mismatch genes for mouse sgRNA *mmKras.366* showed no mutational changes in *KRAS* knockout clones. Potential off-target sequences are highlighted in blue.

c) Total and GTP-bound NRAS and HRAS levels were not significantly changed in *KRAS* knockout clones. A13 cells did not express detectable levels of HRAS protein. Quantitative ratios of total and GTP-bound RAS proteins levels (relative to HSP90 loading control and normalized to the leftmost intact clone) are shown below the corresponding blots.

d) Western blot demonstrated re-expression of oncogenic *KRAS G12V* in knockout clones (T1 and T2). Beta-tubulin is loading control.

e) Re-expression of oncogenic *KRAS G12V* reverted cell morphology similar to that observed in 8988T parental cells. Scale bar is 50 µm.

f) Re-expression of oncogenic *KRAS G12V* enhanced proliferation rate *in vitro* comparable to 8988T parental cells. Average cell viability (normalized to day 0) +/- s.d. (n=5 replicates per cell line per time point) is shown.

g) Re-expression of oncogenic *KRAS G12V* increased soft agar colony formation similar to that observed with 8988T parental cells. Scale bar is 500 µm.

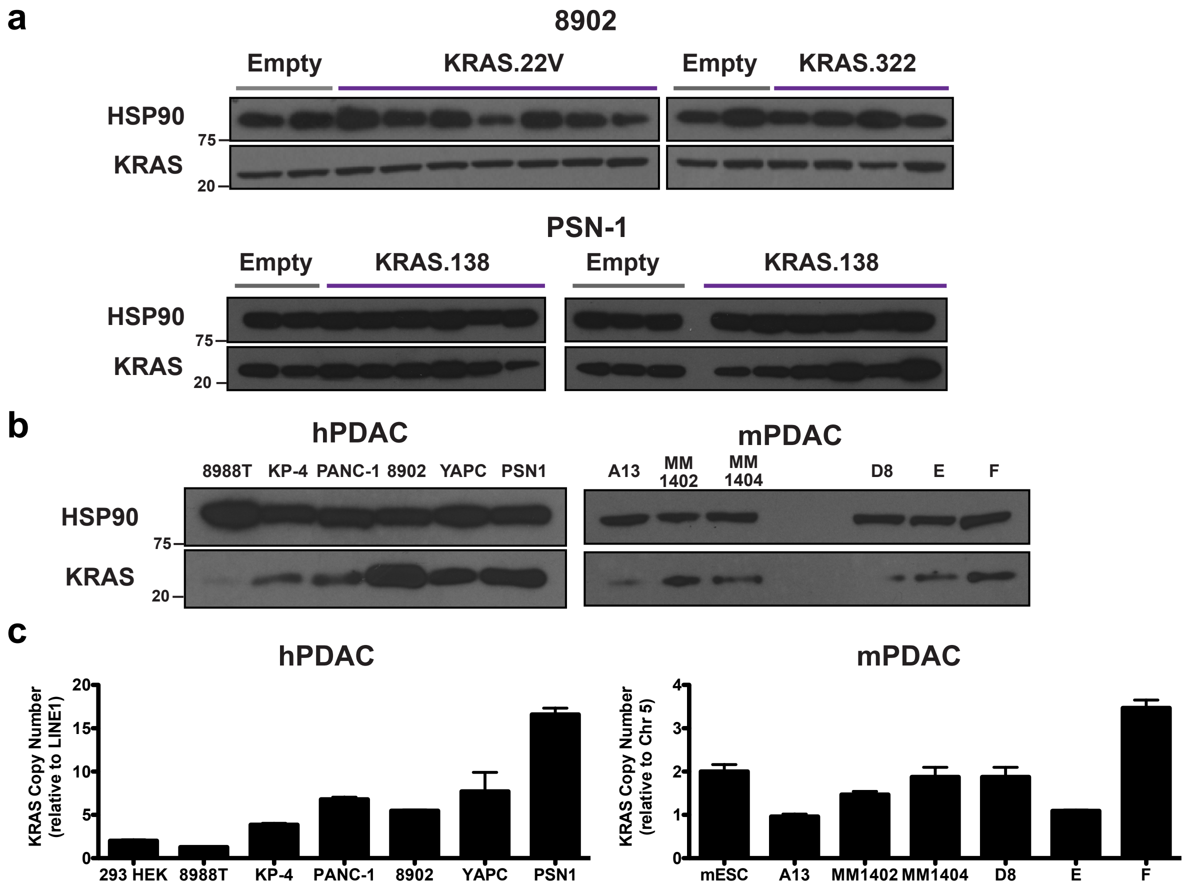

Supplementary Fig. 4. Variable efficiency of *KRAS* knockout clone generation.

a) All 8902 and PSN1 subclones assayed by western blot retained baseline KRAS protein levels (compared to empty vector controls). Purple denotes cells transduced with sgRNAs targeting *KRAS*, while grey specifies clones transduced with empty vector control.

b) Western blot of baseline KRAS protein levels in hPDAC and mPDAC parental cell lines used for CRISPR/Cas-mediate *KRAS* knockout. HSP90 is loading control.

c) *KRAS* copy number in hPDAC and mPDAC parental cell lines relative to 2N controls (293HEK for human cells, murine ES cells (mESC) for mouse cells).

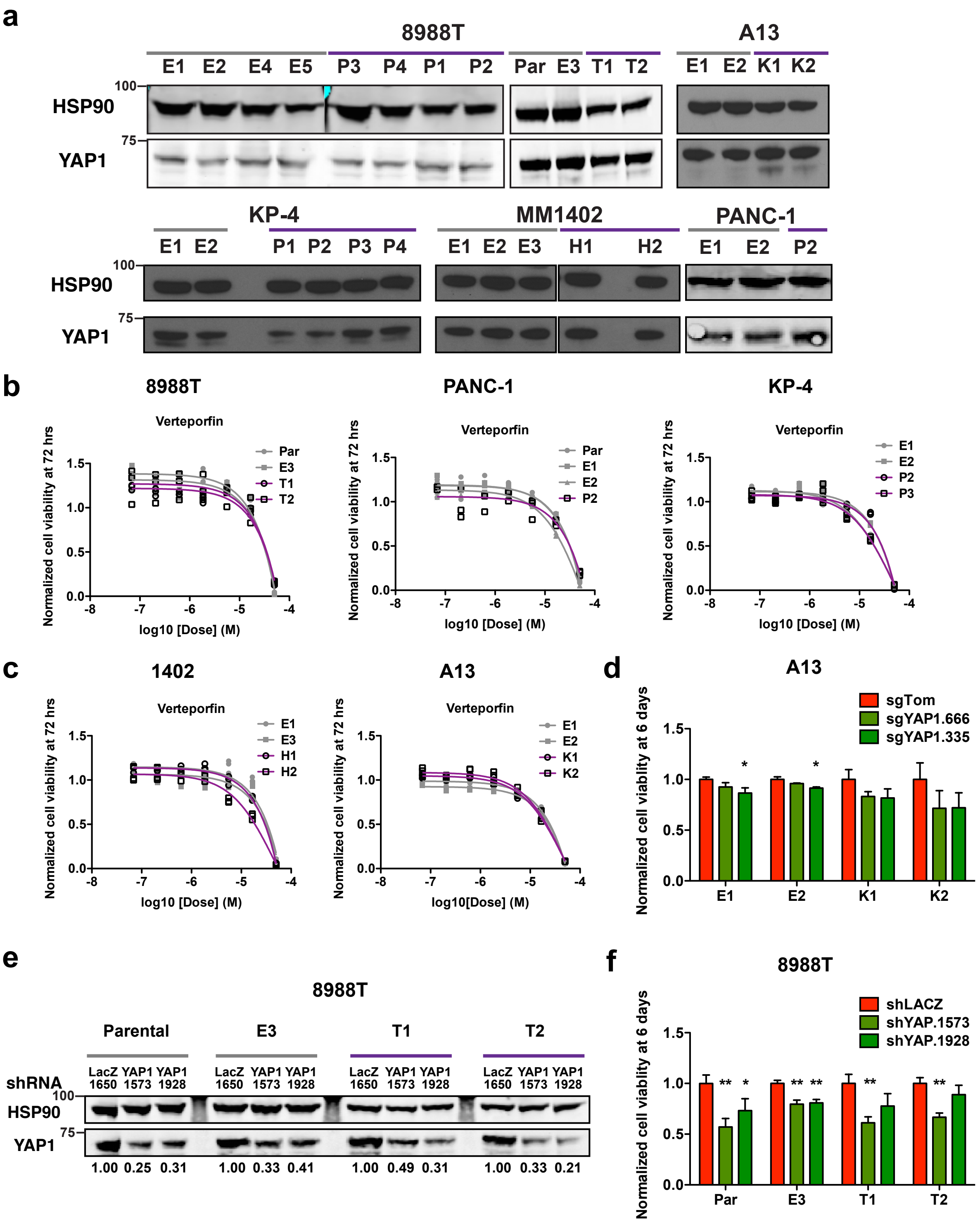

Supplementary Fig. 5. ***KRAS*** **deficient cells are not more dependent on YAP1.**

a) Western blot showed no difference in YAP1 protein levels comparing *KRAS* intact (grey) and *KRAS* deficient (purple) clones derived from 8988T, A13, KP-4, MM1402, and PANC-1 cells. HSP90 is loading control.

b) Dose-response curves of hPDAC intact (grey) and deficient (purple) cells demonstrated comparable sensitivity to the YAP/TEAD interaction inhibitor verteporfin. Each replicate (n=3 for each dose) and curve fit are shown.

c) Dose-response curves of mPDAC *KRAS* intact (grey) and deficient (purple) cells displayed comparable sensitivity to verteporfin. Each replicate (n=3 for each dose) and curve fit are shown.

d) YAP1 knockout did not induce significant differential effects on cell viability comparing A13 *KRAS* intact (E1, E2) and deficient (K1, K2) cells. Average cell viability +/- s.d. (normalized to sgTomato, n=3 replicates per cell line per sgRNA) is shown. *p<0.01 comparing sgYAP1 to sgTomato control, two-tailed Student's t-test.

e) Western blot showed YAP1 protein knockdown in 8988T *KRAS* intact (grey) and deficient (purple) cells transduced with shRNAs targeting YAP1 or shLacZ control. Quantitative ratios of YAP1 (normalized to HSP90 loading control) relative to shLacZ are shown.

f) YAP1 knockdown did not induce significant differential effects on cell viability comparing 8988T *KRAS* intact (8988T parental (Par), E3) and deficient (T1, T2) cells. Average cell viability +/- s.d. (normalized to shLACZ, n=3 replicates per cell line per shRNA) is shown. **p<0.01, *p<0.01 comparing shYAP1 to shLACZ control, two-tailed Student's t-test.

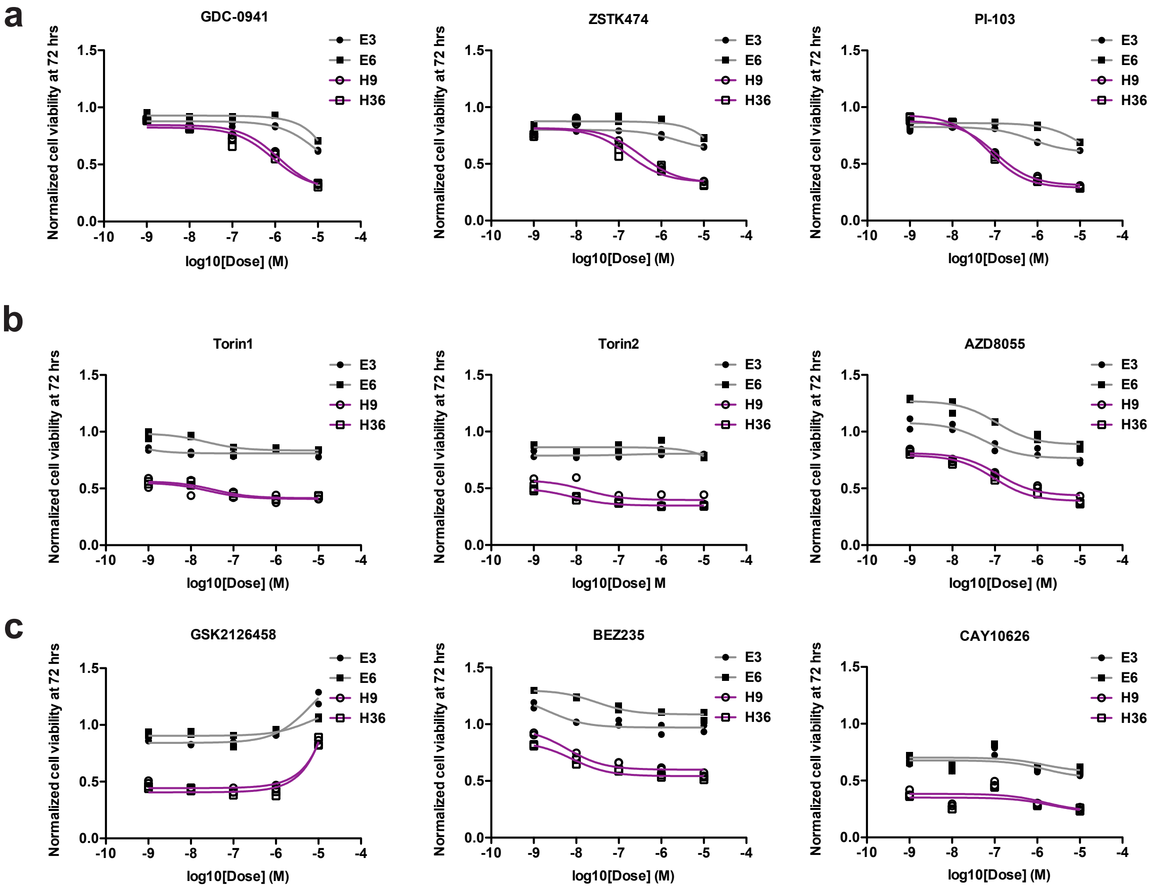

Supplementary Fig. 6. PI3K and MTOR dependence of *KRAS* deficient cells.

a) Dose-response curves for pan-PI3K inhibitors in compound library on intact and knockout cells. Each replicate (n=3 for each dose) and curve fit are shown.

b) Dose-response curves for mTOR inhibitors in compound library. Each replicate (n=3 for each dose) and curve fit are shown.

c) Dose-response curves for PI3K/mTOR inhibitors in compound library. Each replicate (n=3 for each dose) and curve fit are shown.

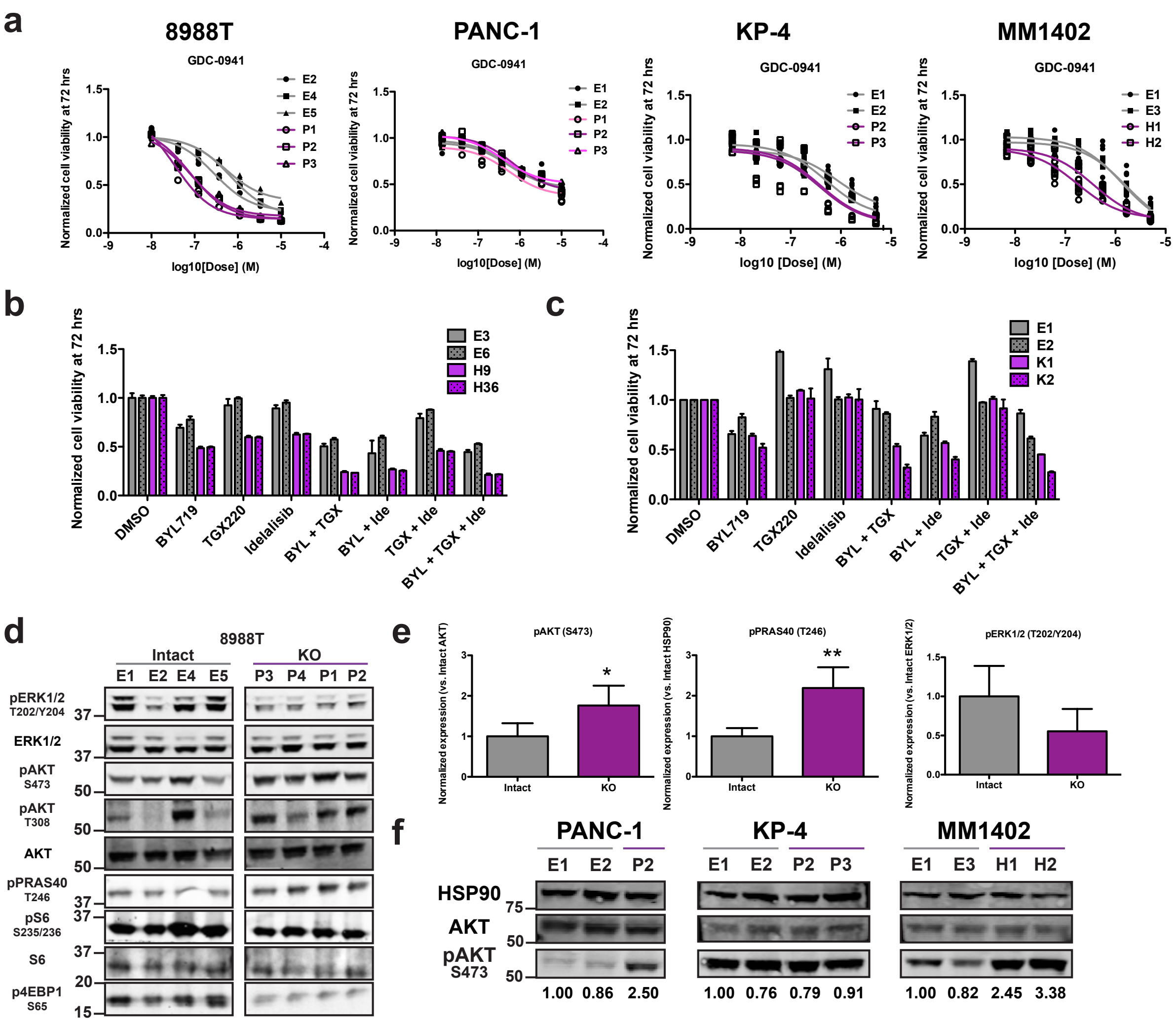

Supplementary Fig. 7. PI3K pathway activation and dependence in *KRAS* deficient cells.

a) Dose-response curves of additional 8988T, PANC-1, KP-4, and MM1402 intact (grey) and deficient (purple) clones to GDC-0941. Each replicate (n=3-6 for each dose) and curve fit are shown.

b) Average cell viability (normalized to DMSO solvent, n=3 replicates per cell line per condition) +/- s.e.m. of 8988T *KRAS* intact (E3 and E6) and deficient (H9 and H36) clones after treatment with various combinations of p110α (BYL719 = BYL), p110β (TGX220 = TGX), and p110δ (Idelalisib = Ide) inhibitors for 72 hours. All inhibitors alone or in combination were given at 5 μM concentration.

c) Average cell viability (normalized to DMSO solvent, n=3 replicates per cell line per condition) +/- s.e.m. of A13 *KRAS* intact (E1 and E2) and deficient (K1 and K2) clones after treatment with isoform-specific p110 inhibitors for 72 hours as in (b).

d) Western blots showed activation of the MAPK (pERK1/2) and PI3K/AKT (pAKT, pPRAS40, pS6, p4EBP1) pathways in 8988T *KRAS* intact and deficient (KO) clones.

e) Quantitation of pAKT (normalized to total AKT), pPRAS40 (normalized to HSP90 loading control), and pERK1/2 (normalized to total ERK1/2) levels +/- s.d. in 8988T clones (n=5-6 clones per group). *$p<0.05$, **$p<0.01$, Mann-Whitney U-Test.

f) Western blot showed increased pAKT levels in PANC-1 and MM1402, but not KP-4, *KRAS* deficient cells consistent with PI3K/AKT pathway activation. Grey lines denote *KRAS* intact clones and purple lines designate deficient clones. Quantitative ratios of pAKT/AKT (normalized to leftmost intact clone) are shown.

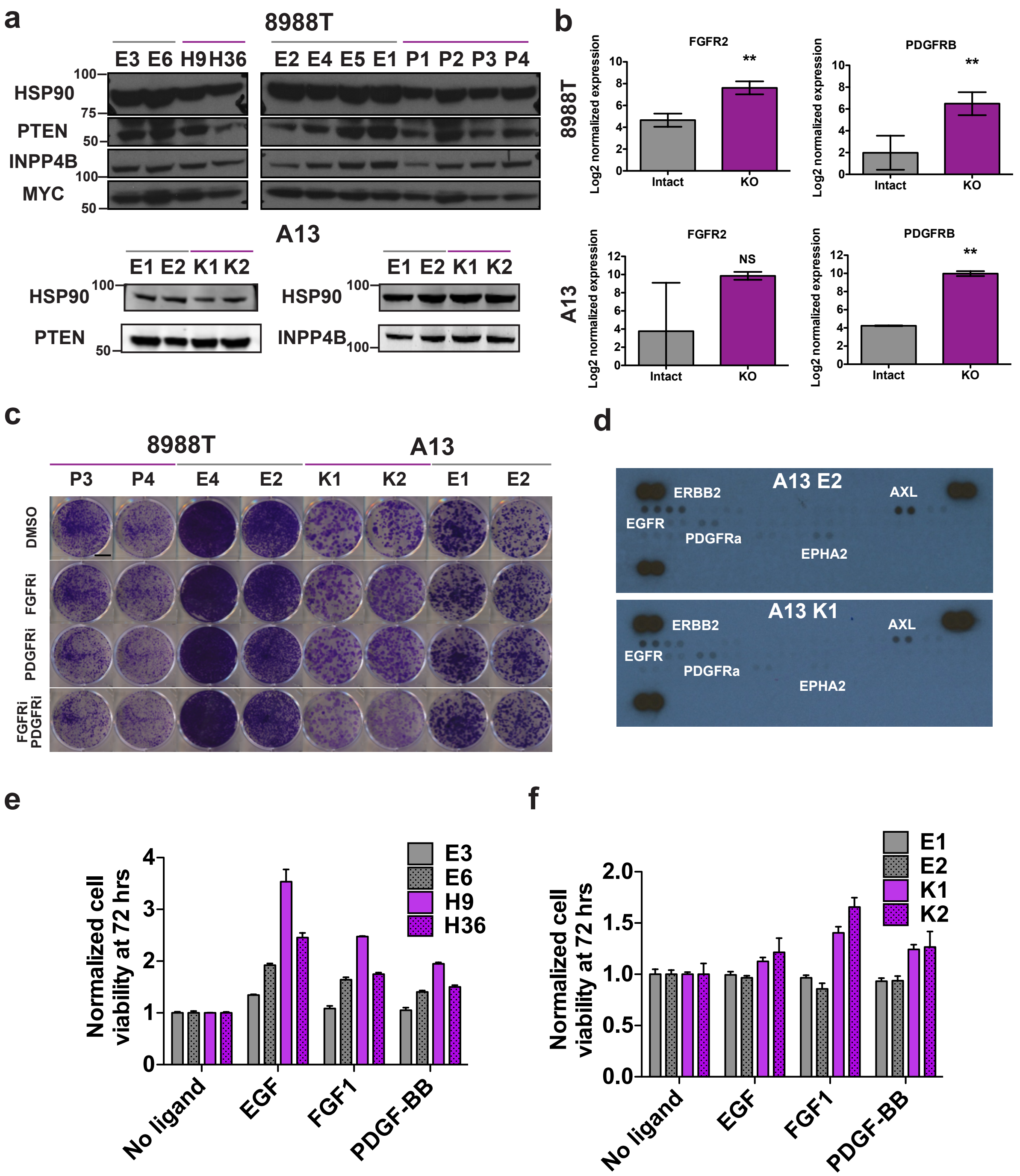

Supplementary Fig. 8. Evaluation of potential mechanisms for increased PI3K/AKT activation in *KRAS* deficient cells

a) Western blots showed no significant consistent change in PTEN, INPP4B, and MYC levels between *KRAS* intact (grey) and deficient (purple) clones. HSP90 is loading control.

b) Normalized *FGFR2* and *PDGFRβ* gene expression (log2) +/- s.d. by RNA-Seq in 8988T (n=3-4 clones in each group) and A13 cells (n=2 clones in each group). ** $p<0.01$, two-tailed Student's t-test.

c) Crystal violet staining following 10 days of treatment with designated inhibitors showed no significant differences between 8988T and A13 *KRAS* intact (grey) and deficient (purple) clones. FGFRi = 100 nM BGJ398, PDGFRi = 100 nM crenolanib. Control is DMSO solvent. Scale bar is 1 cm.

d) RTK array profiling of *KRAS* intact (A13-E2) and deficient (A13-K1) clones showed no differential activation of RTKs. Similar results were observed for comparison of A13-E1 and A13-K2.

e) Average cell viability (normalized to no ligand) +/- s.e.m. (n=3 replicates per cell line per condition) of 8988T *KRAS* intact (grey shades) and deficient (purple shades) clones after 72 hours of treatment with 2 µM GDC-0941 and designated recombinant human ligands: 20 nM epidermal growth factor (EGF), 100 ng/mL fibroblast growth factor 1 (FGF1), or 100 ng/mL platelet-derived growth factor BB (PDGF-BB).

f) Average cell viability (normalized to no ligand) +/- s.e.m. (n=3 replicates per cell line per condition) of A13 *KRAS* intact (grey shades) and deficient (purple shades) clones after 72 hours of treatment with 2 µM GDC-0941 and designated recombinant human ligands in (e).

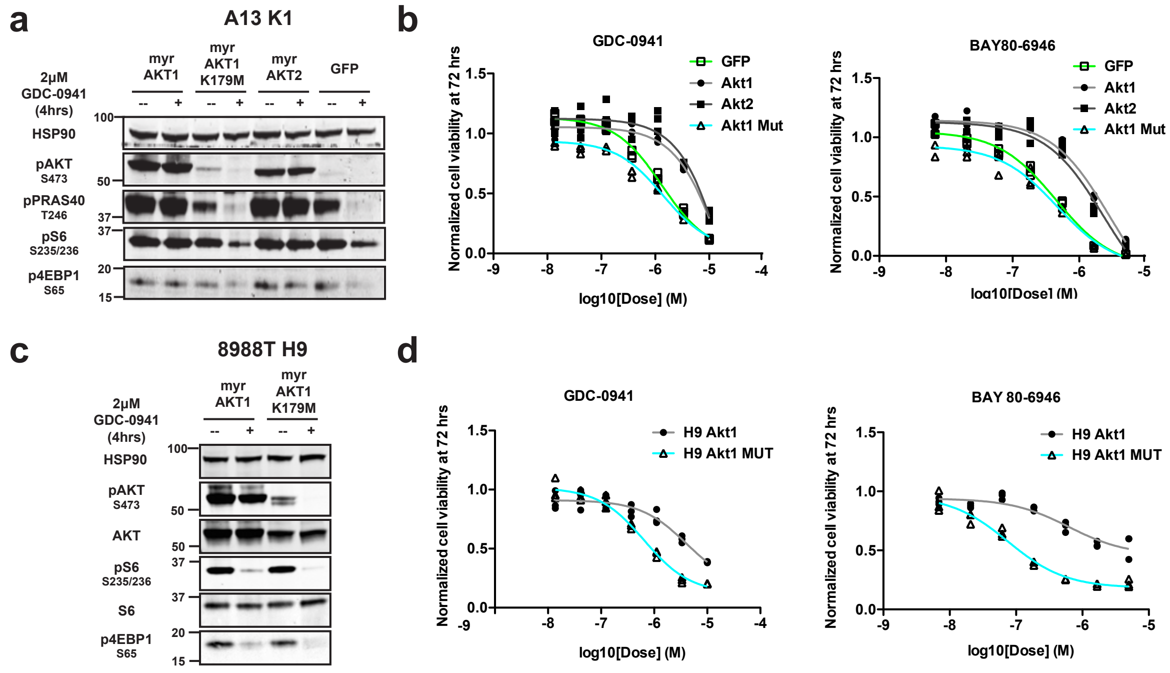

Supplementary Fig. 9. PI3K inhibition functions through AKT–dependent mechanisms.

a) Western blot revealed sustained phosphorylation of AKT and downstream targets (PRAS40, S6, and 4EBP1) in A13 K1 *KRAS* deficient cells overexpressing *myr-AKT1* or *myr-AKT2* but not kinase-dead *myr-AKT1 (K179M)* (*Akt1 Mut*) or *GFP* following a 4-hour treatment with 2µM GDC-0941.

b) Dose-response curves of cell lines in (a) treated with GDC-0941 and BAY80-6946 showed a marked decrease in PI3K inhibitor sensitivity with *myr-AKT1* or *myr-AKT2* overexpression but not with *myr-AKT1 (K179M)* compared to *GFP* control.

c) Western blot showed maintained phosphorylation of AKT and downstream targets (PRAS40, S6, and 4EBP1) in 8988T H9 *KRAS* deficient cells overexpressing *myr-AKT1* but not kinase-dead *myr-AKT1 (K179M)* (*Akt1 Mut*) after a 4-hour treatment with 2µM GDC-0941.

d) Dose-response curves of cell lines in (c) treated with GDC-0941 and BAY80-6946 revealed a marked decrease in PI3K inhibitor sensitivity with *myr-AKT1* overexpression compared to kinase-dead control.

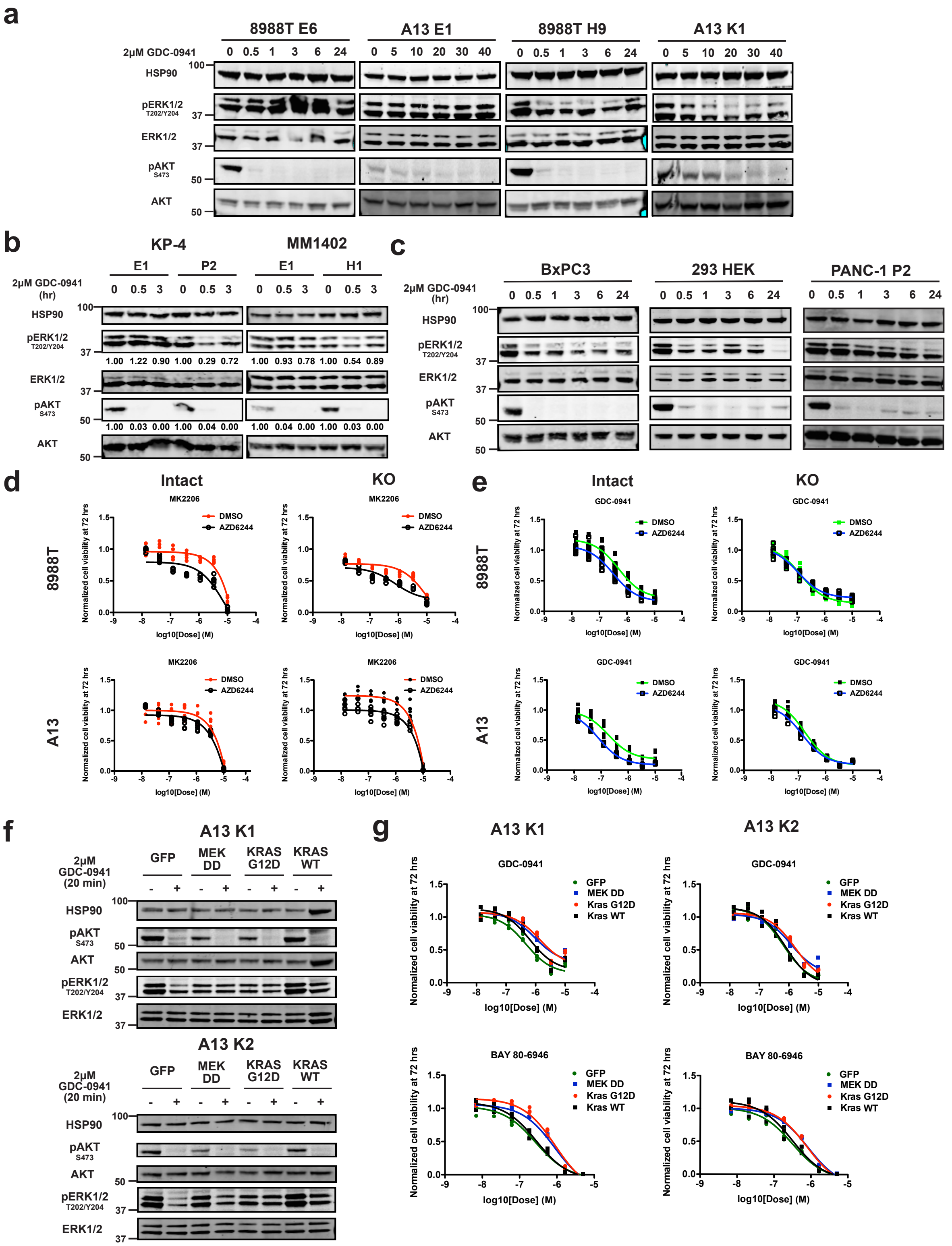

Supplementary Fig. 10. MAPK pathway blockade mediates anti-growth effects of PI3K inhibition in *KRAS* deficient cells.

a) Western blot showed no change in pERK1/2 levels in additional A13 and 8988T *KRAS* intact (8988T-E6, A13-E1) and deficient (8988T-H9, A13-K1) cells at designated times (minutes for A13, hours for 8988T) following GDC-0941 treatment. HSP90 is loading control.

b) Western blot showed a transient decrease in pERK1/2 levels in *KRAS* deficient (KP-4-P2, MM1402-H1) but not intact (KP-4-E1, MM1402-E1) clones derived from KP-4 and MM1402 cell lines following GDC-0941 treatment. Quantitative ratios of pAKT (normalized to total AKT) and pERK1/2 (normalized to total ERK1/2) are shown below the corresponding blots.

c) Western blot showed a transient decrease in pERK1/2 levels in the *KRAS* wild type cell line BxPC3 and human embryonic kidney (293 HEK) cells but not PANC-1 *KRAS* deficient cells (P2) following GDC-0941 treatment.

d) Dose-response curves of *KRAS* intact or deficient (KO) cell lines treated with the pan-AKT inhibitor MK2206 and the MEK inhibitor AZD6244 (2μM) (black) or DMSO (red). Each replicate (n=6 for each dose) and curve fit are shown.

e) Dose-response curves of *KRAS* intact or deficient (KO) cell lines treated with GDC-0941 and 2μM AZD6244 (blue) or DMSO (green). Each replicate (n=6 for each dose) and curve fit are shown.

f) Western blot revealed that overexpression of constitutively active MEK (*MEK-DD*) or oncogenic *Kras G12D*, but not *Kras WT* or *GFP*, blocked pERK1/2 inhibition by GDC-0941 in A13 deficient cells.

g) *MEK-DD* and *Kras G12D*-transduced cells from (f) showed decreased sensitivity to PI3K inhibition (GDC-0941 and BAY80-6946) compared to control *GFP*- and *Kras WT*-transduced cells.

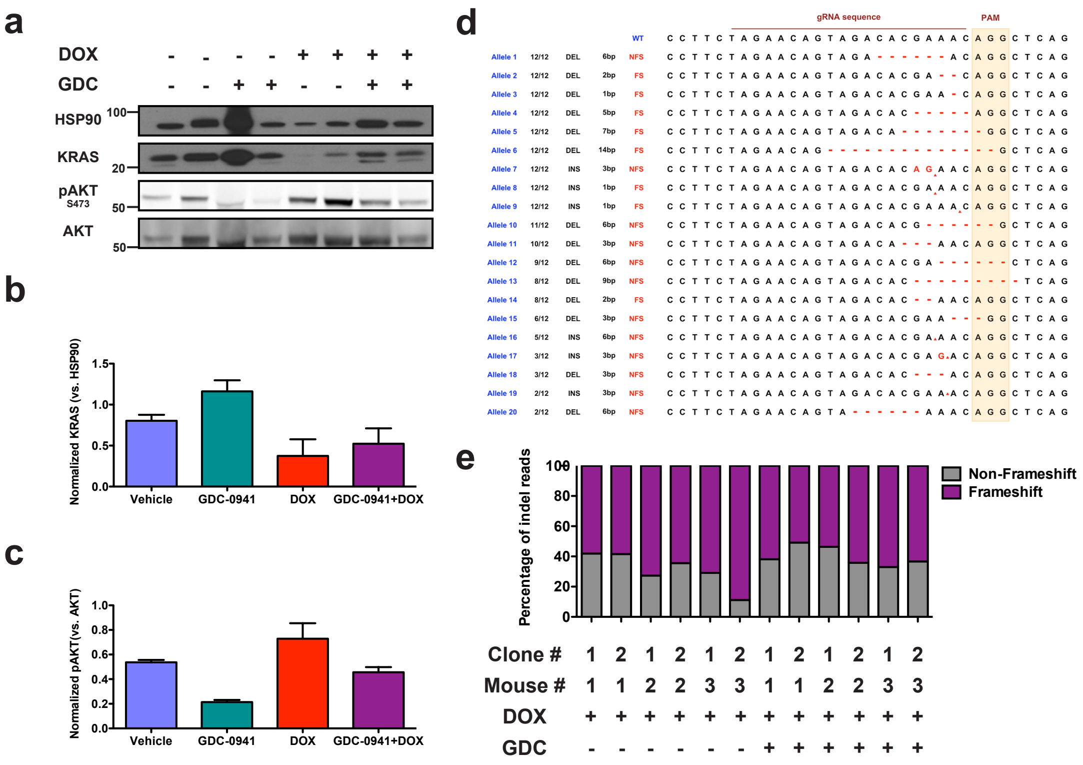

Supplementary Fig. 11. Combined KRAS and PI3K inhibition in established PDAC tumors.

a) Western blots of KRAS, pAKT (S473), and AKT protein levels in tumors derived from mice subject to KRAS and/or PI3K inhibition. HSP90 is loading control. GDC = GDC-0941.

b) Quantitation of KRAS protein levels (normalized to HSP90 loading control, n=4 tumors per group, 2 per clone) +/- s.e.m. showed decreased KRAS levels in tumors from DOX-treated mice consistent with induction of *KRAS* knockout. In addition, combined inhibition with DOX and GDC-0941 led to an increase in KRAS protein levels compared to DOX alone.

c) Quantitation of pAKT (S473) protein levels (normalized to total AKT, n=4 tumors per group, 2 per clone) +/- s.e.m. showed decreased pAKT levels in tumors derived from GDC-0941-treated mice. In addition, we observed increased pAKT levels in tumors from DOX-treated mice consistent with induction of PI3K/AKT pathway activation following *KRAS* knockout.

d) Alignments of top twenty *KRAS* mutant alleles observed in multiple samples compared to the wild-type (WT) locus sequence. Locations of the guide RNA target sequence and PAM site are indicated. Each mutant allele is characterized by its frequency across all samples (n=12), the type of event (DEL = deletion, INS = insertion), event size (in bp), and impact on coding sequence (FS = Frame-shift, NFS = Non-Frame-shift). Mismatches are shown in red, while deletions are shown as red dashes and insertions as red triangles.

e) *KRAS* non-frameshift and frameshift indel allele fractions in tumors derived from mice subject to KRAS and/or PI3K inhibition.

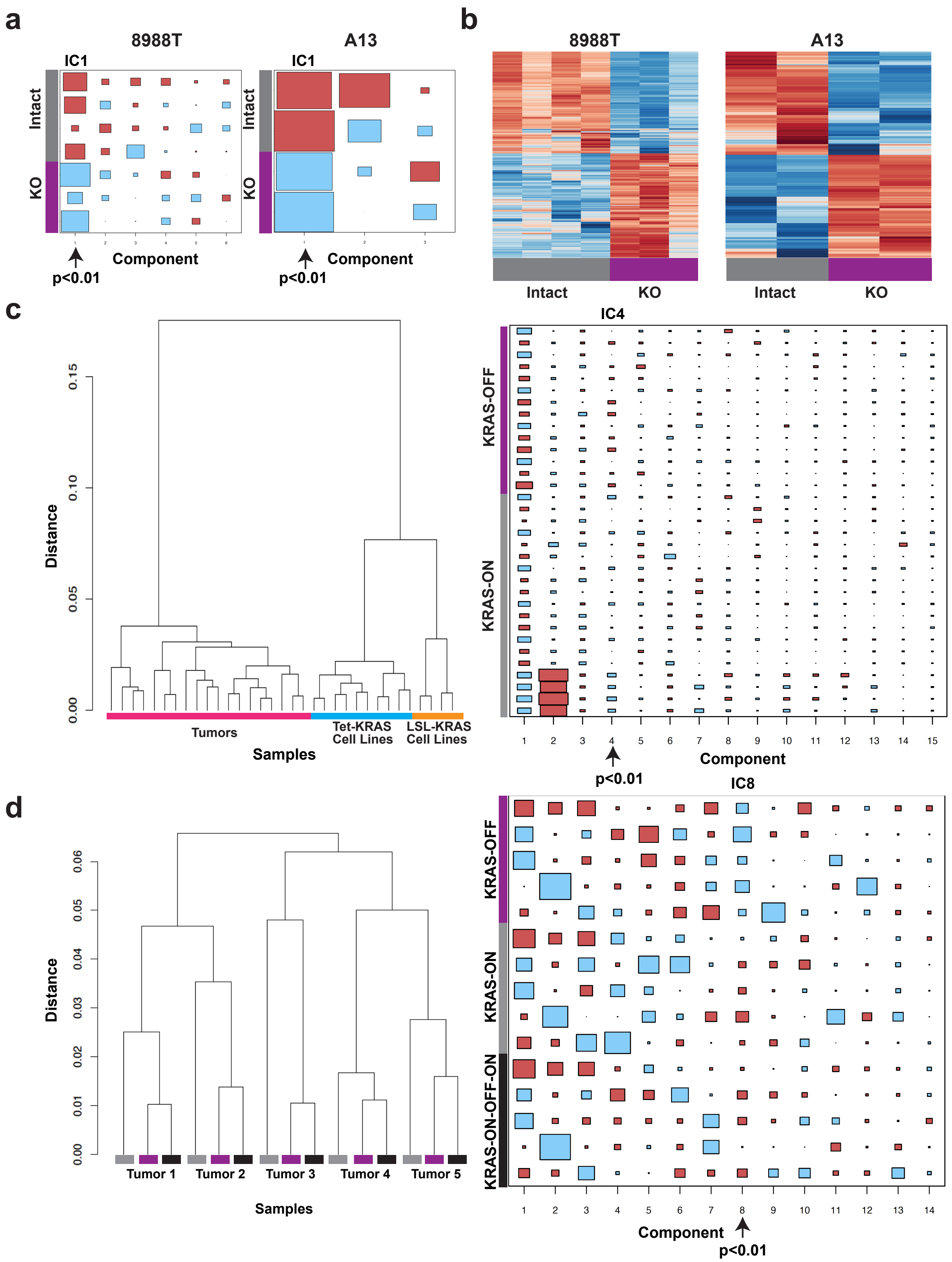

Supplementary Fig. 12. ICA-derived gene expression signatures from 8988T and A13 cells and external datasets derived from a *KRAS* transgenic model.

a) Hinton diagrams of ICA analyses of 8988T and A13 *KRAS* intact and deficient (KO) clones. Columns represent distinct gene expression patterns (signatures) where colors encode directionality of gene expression (red relatively upregulated, blue relatively downregulated within each signature component). Sizes of individual boxes correlate with strength of association between each signature and a given sample (row). Independent component 1 (IC1) distinguished intact and knockout clones ($p<0.01$, Mann-Whitney U-Test) in each case.
b) Heatmaps of ICA-derived signatures comprising the top 2% upregulated and top 2% downregulated genes (FC>2, rows) in A13 and 8988T intact and deficient (KO) clones (columns). Row normalized gene expression values are shown where red designates upregulation and blue designates downregulation.
c) Unsupervised hierarchical clustering of microarray data from Ying et al.[1] from tumors and cell lines in which oncogenic *KRAS* expression was acutely withdrawn in transgenic animals. Samples segregated based on origin (tumors vs. cell lines) and model system (transgenic Tet-KRAS cell lines vs. *LSL-KRAS* cell lines) and not on KRAS-OFF or KRAS-ON state. ICA analysis identified a signature (component 4 (IC4) in Hinton diagram) that distinguished KRAS-OFF and KRAS-ON cells ($p<0.01$, Mann-Whitney U-Test).
d) Unsupervised hierarchical clustering of microarray data from Viale et al.[2] using cells derived from tumors following *KRAS* transgene withdrawal. Samples segregated on tumors and not on KRAS-OFF or KRAS-ON state. ICA analysis identified a signature (component 8 (IC8) in Hinton diagram) that distinguished KRAS-OFF and KRAS-ON or KRAS-ON-OFF-ON (oncogenic *KRAS* withdrawn and then re-expressed) tumor cells ($p<0.01$, Mann-Whitney U-Test).

a

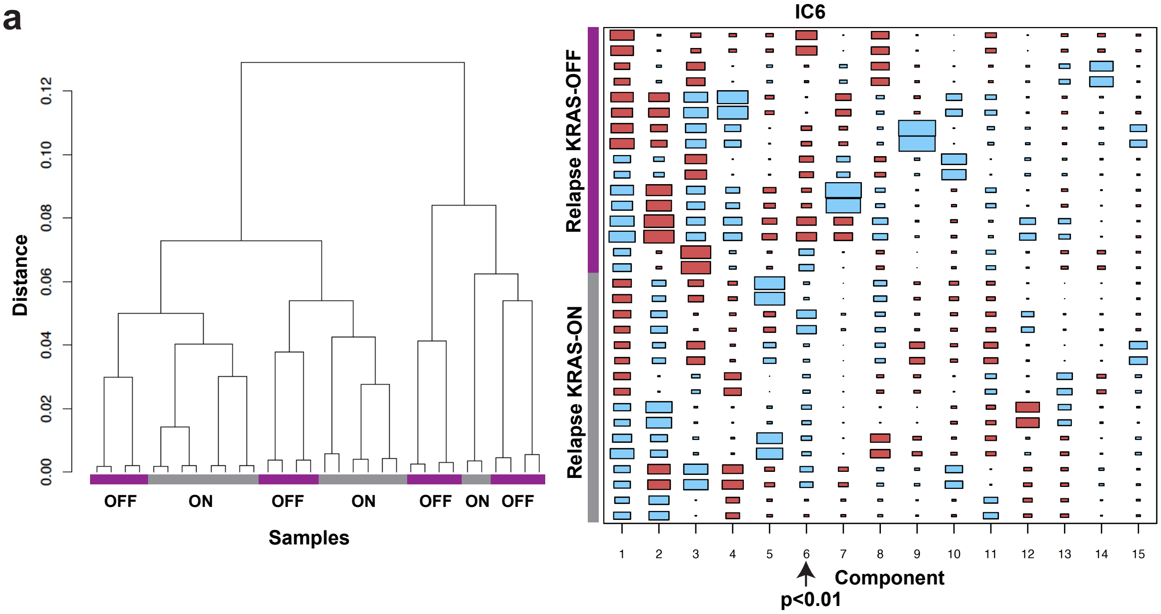

b

8988T KO signature

Gene Set	NES	p-value
YING IC4 KRAS OFF	1.98	0
KAPOOR IC6 KRAS OFF	1.54	0.015
KAPOOR IC6 KRAS ON	1.50	0.022
VIALE IC8 KRAS OFF	1.46	0.051
YING IC4 KRAS ON	1.31	0.091
VIALE IC8 KRAS ON	-1.41	0.042

A13 KO signature

Gene Set	NES	p-value
YING IC4 KRAS OFF	2.27	0
VIALE IC8 KRAS OFF	2.20	0
KAPOOR IC6 KRAS OFF	1.98	0
VIALE IC8 KRAS ON	-2.14	0
KAPOOR IC6 KRAS ON	-2.13	0
YING IC4 KRAS ON	-1.59	0.002

Supplementary Fig. 13. Comparison of *KRAS* knockout gene signatures with external datasets derived from a *KRAS* transgenic model.

a) Unsupervised hierarchical clustering of microarray data from Kapoor et al.[3] using cell lines derived from relapsed tumors following *KRAS* withdrawal, which continued to express oncogenic *KRAS* (KRAS-ON) or did not (KRAS-OFF). Samples did not cleanly segregate based on KRAS-ON or KRAS-OFF status. ICA analysis identified a signature (component 6 (IC6) in Hinton diagram) that distinguished relapsed KRAS-OFF and relapsed KRAS-ON tumor cell lines ($p<0.01$, Mann-Whitney U-Test).

b) GSEA revealed enrichment of KRAS-OFF signatures from **Supplementary Figs. 12c, 12d, and 13a** in 8988T and A13 knockout signatures. A13 also exhibited statistically significant anti-correlation with KRAS-ON signatures from these external datasets.

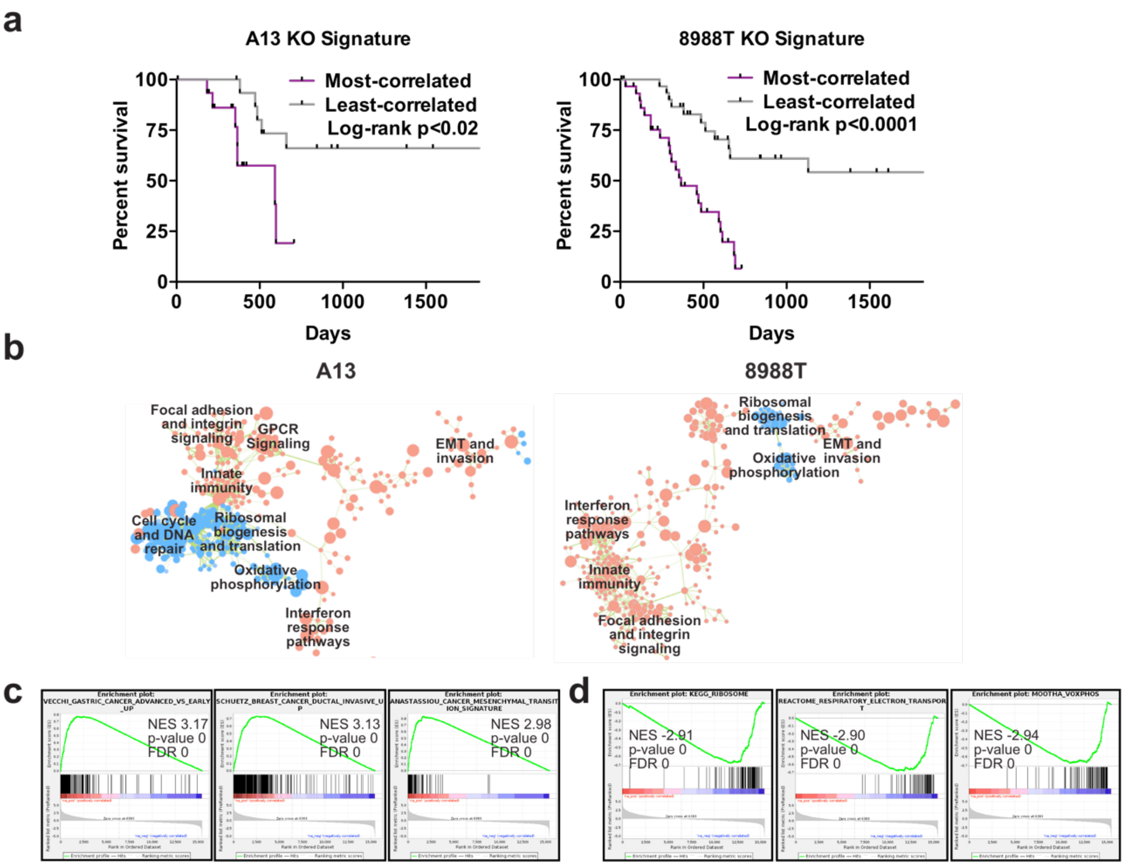

e

8988T sig.

Characteristic	Univariate Analysis HR (95% CI)	Univariate p-value	Multivariable Analysis HR (95% CI)	Multivariable p-value	$p_{interaction}$
KRAS KO (8988T sig.)	2.47 (1.61-3.78)	**0.00004**	3.15 (1.62-6.15)	**0.000762**	-
Gender (Male vs Female)	1.07 (0.53-2.19)	0.84736	0.70 (0.25-1.95)	0.491752	
Age (Years)	1.03 (0.99-1.06)	0.15462	1.04 (0.99-1.09)	0.089449	
T score (T3 vs T1/T2)	7.05 (1.65-30.08)	**0.00831**	1.19 (0.22-6.54)	0.84062	
N score (N1 vs N0)	3.27 (1.22-8.72)	**0.01809**	0.37 (0.10-1.43)	0.149531	
Completeness of resection (R1 vs R0)	2.14 (1.02-4.50)	**0.04492**	1.37 (0.55-3.46)	0.4995	
Number of lymph nodes	1.02 (0.95-1.11)	0.54742	1.11 (0.94-1.30)	0.215673	

A13 sig.

Characteristic	Univariate Analysis HR (95% CI)	Univariate p-value	Multivariable Analysis HR (95% CI)	Multivariable p-value	$p_{interaction}$
KRAS KO (A13 sig.)	1.63 (1.04-2.55)	**0.03295**	2.55 (1.20-5.45)	**0.0152**	-
Gender (Male vs Female)	1.82 (0.49-6.75)	0.36886	4.80 (0.71-32.38)	0.1074	
Age (Years)	1.00 (0.94-1.07)	0.93204	1.03 (0.92-1.15)	0.5721	
T score (T3 vs T1/T2)	5.53 (0.70-43.55)	0.10464	11.47 (0.47-277.44)	0.1335	
N score (N1 vs N0)	3.06 (0.76-12.23)	0.11398	1.79 (0.21-15.00)	0.5933	
Completeness of resection (R1 vs R0)	0.98 (0.21-4.58)	0.98359	0.29 (0.04-2.28)	0.2404	
Number of lymph nodes	1.03 (0.91-1.17)	0.59941	1.20 (0.88-1.63)	0.2449	

Combined sig.

Characteristic	Univariate Analysis HR (95% CI)	Univariate p-value	Multivariable Analysis HR (95% CI)	Multivariable p-value	$p_{interaction}$
KRAS KO (combined sig.)	2.18 (1.44-3.29)	**0.00023**	4.70 (1.73-12.77)	**0.00246**	-
Gender (Male vs Female)	1.41 (0.61-3.23)	0.41738	2.48 (0.83-7.37)	0.1022	
Age (Years)	1.06 (1.02-1.11)	**0.0081**	1.05 (1.01-1.09)	**0.02604**	0.05349
T score (T3 vs T1/T2)	6.38 (1.47-27.61)	**0.01321**	5.01 (0.69-36.32)	0.11112	
N score (N1 vs N0)	5.12 (1.50-17.50)	**0.0092**	0.63 (0.12-3.40)	0.59487	
Completeness of resection (R1 vs R0)	3.03 (1.31-7.01)	**0.00949**	2.63 (0.82-8.44)	0.10526	
Number of lymph nodes	1.06 (0.98-1.15)	0.12783	1.12 (0.95-1.33)	0.18297	

HR = Hazard ratio; CI = Confidence Interval; T score = Primary tumor size/invasiveness; N score = Lymph node metastasis

$p_{interaction}$ = p-value of interaction between significant covariates (likelihood ratio test)

Supplementary Fig. 14. Human PDAC tumors correlating with *KRAS* knockout signatures are associated with worse survival.

a) Kaplan-Meier plots of survival of patients in TCGA cohort whose tumors most-correlated or least-correlated with the A13 (n=16 most-correlated and n=17 least-correlated tumors) and 8988T (n=33 most-correlated and n=33 least-correlated tumors) knockout signatures. Log-rank (Mantel-Cox) p-values are shown.
b) Network representation of overlapping enriched GSEA/MSigDB gene sets in human tumors from TCGA most correlated with the A13 and 8988T knockout signature ($p<0.05$, $FDR<0.25$).
c) Significantly enriched GSEA/MSigDB gene sets in human tumors from TCGA most-correlated with the 8988T and A13 knockout signatures included those associated with advanced disease, EMT, and invasion. Normalized enrichment scores (NES), p-values, and FDR are listed with relation to the A13 knockout signature.
d) Significantly enriched GSEA/MSigDB gene sets in human tumors from TCGA least-correlated with the 8988T and A13 knockout signatures included those associated with ribosome and oxidative phosphorylation. Normalized enrichment scores (NES), p-values, and FDR are listed with relation to the A13 knockout signature.
e) Univariate and multivariable Cox proportional hazards models on overall survival of tumors/patients in the TCGA cohort which were most-correlated (top quintile) or least-correlated (bottom quintile) with the combined *KRAS* knockout signature. Hazard ratios (HR) and p-values (Cox regression) are reported. A comparison between a model with and without an interaction term (likelihood ratio test) was used to determine independence ($p>0.05$) between significant covariates in multivariable analysis and the combined *KRAS* knockout signature.

Supplementary Table 1. sgRNA and shRNA sequences targeting human and mouse genes

sgRNA	20 bp Guide Sequence	PAM	Orientation	Species	Exon	Protein Decrease	Source
hsKRAS.22V	GTAGTTGGAGCTGTTGGCGT	AGG	Sense	Human	1	Y	Designed
hsKRAS.22D	GTAGTTGGAGCTGATGGCGT	AGG	Sense	Human	1	N	Designed
hsKRAS.75	CTGAATTAGCTGTATCGTCA	AGG	Antisense	Human	1	N	crispr.mit.edu
hsKRAS.138	AATTACTACTTGCTTCCTGT	AGG	Antisense	Human	2	Y	Designed
hsKRAS.165	TCTCGACACAGCAGGTCAAG	AGG	Sense	Human	2	Y	GeCKO v2
hsKRAS.197	CAATGAGGGACCAGTACATG	AGG	Sense	Human	2	Y	GeCKO v2
hsKRAS.312	GGACTCTGAAGATGTACCTA	TGG	Sense	Human	3	N	crispr.mit.edu
hsKRAS.322	GATGTACCTATGGTCCTAGT	AGG	Sense	Human	3	Y	GeCKO v2
hsKRAS.391	CAGGACTTAGCAAGAAGTTA	TGG	Sense	Human	3	N	GeCKO v2
hsKRAS.486	TTCTCGAACTAATGTATAGA	AGG	Antisense	Human	4	N	GeCKO v2
mmKras.75	CTGAATTAGCTGTATCGTCA	AGG	Antisense	Mouse	1	N	crispr.mit.edu
mmKras.157	TTGGATATTCTCGACACAGC	AGG	Sense	Mouse	2	Y	crispr.mit.edu
mmKras.171	GTCGAGAATATCCAAGAGAC	AGG	Antisense	Mouse	2	Y	crispr.mit.edu
mmKras.366	TAGAACAGTAGACACGAAAC	AGG	Sense	Mouse	3	Y	crispr.mit.edu
mmKras.318	TGAAGATGTGCCTATGGTCC	TGG	Sense	Mouse	3	Y	crispr.mit.edu
mmKras.138	AATTACTACTTGTTTCCTGT	AGG	Antisense	Mouse	2	Y	Designed
Tomato	GGCCACGAGTTCGAGATCGA	GGG	Sense	Coral	N/A	Y	Designed
mmYAP1.666	GATGATGTACCACTGCCAGC	AGG	Sense	Mouse	2	Y	Designed
mmYAP1.335	CGGGGACTCGGAGACCGACT	TGG	Sense	Mouse	1	Y	Designed
**shRNA**	**Target Sequences**	**Species**	**Source**				
shLacZ.1650	CGCTAAATACTGGCAGGCGTT	Human	Gift from William Hahn				
shKRAS.407	GAGGGCTTTCTTTGTGTATTT	Human	Gift from William Hahn				
shYAP1.1573	GCCACCAAGCTAGATAAAGAA	Human	Gift from William Hahn				
shYAP1.1928	CCCAGTTAAATGTTCACCAAT	Human	Gift from William Hahn				

Source Key

Designed = self-designed based on available PAM sequences

crispr.mit.edu = sequences outputted from Feng Zhang lab CRISPR design tool

GeCKO v2 = sequences from GeCKO v2 library from Feng Zhang lab

Supplementary Table 2. PDAC cell lines

Cell Line	Species	Source	Cell Line Type	*KRAS* Genotype	Culture Media	Capable of generating *KRAS* knockouts
8988T	Human	Broad CCLE (sourced from DSMZ)	Established	G12V	DMEM/10% FBS	Yes
PANC-1	Human	Broad CCLE (sourced from ATCC)	Established	G12D	DMEM/10% FBS	Yes
KP-4	Human	Broad CCLE (sourced from ATCC)	Established	G12D	DMEM/10% FBS	Yes
8902	Human	Broad CCLE (sourced from RIKEN)	Established	G12V	DMEM/10% FBS	No
YAPC	Human	Broad CCLE (sourced from DSMZ)	Established	G12V	DMEM/10% FBS	No
PSN1	Human	Broad CCLE (sourced from ATCC)	Established	G12A	RPMI/10% FBS	No
BxPC3	Human	Broad CCLE (sourced from ATCC)	Established	WT	RPMI/10% FBS	Not tested
PACO9	Human	HI-STEM	Primary	G12V	PACO medium	Yes
PACO19	Human	HI-STEM	Primary	G12V	PACO medium	Yes
A13	Mouse	*LSL-Kras^{G12D}; p53$^{flox/flox}$; Pdx1-CreER*	Established	G12D	DMEM/10% FBS	Yes
MM1402	Mouse	*LSL-Kras^{G12D}; p53$^{R172H/WT}$; Pdx1-Cre*	Primary	G12D	DMEM/10% FBS	Yes
MM1404	Mouse	*LSL-Kras^{G12D}; p53$^{R172H/WT}$; Pdx1-Cre*	Primary	G12D	DMEM/10% FBS	No
D8	Mouse	*LSL-Kras^{G12D}; p53$^{flox/flox}$; Pdx1-CreER*	Established	G12D	DMEM/10% FBS	No
E	Mouse	*LSL-Kras^{G12D}; p53$^{flox/flox}$; Pdx1-CreER*	Primary	G12D	DMEM/10% FBS	No
F	Mouse	*LSL-Kras^{G12D}; p53$^{flox/flox}$; Pdx1-CreER*	Primary	G12D	DMEM/10% FBS	No

Supplementary Table 3. Efficiency of *KRAS* deficient clone generation from PDAC cell lines. Single cells were plated in 96-well plates and the number of clones that grew out was quantitated. Numbers of clones screened for knockout (KO) by western blot (WB) and those confirmed to have no protein are listed. PACO9 and PACO19 cells clones were generated by low-density plating in larger culture dishes rather than plating in 96-well plates.

**8988T**	**Wells Plated**	**Grown Out**	**Screened for KO by WB**	**Confirmed KO by WB**
**Empty**	480	28	9	0
hsKRAS.22V	480	36	24	**23***
hsKRAS.165	480	39	39	**2**

***one had protein and no mutagenesis at DNA level (designated E1)**

**PANC-1**	**Wells Plated**	**Grown Out**	**Screened for KO by WB**	**Confirmed KO by WB**
**Empty**	288	16	4	0
hsKRAS.165	288	2	2	**1 partial**
hsKRAS.322	288	4	4	**1 partial, 1 complete**

**8902**	**Wells Plated**	**Grown Out**	**Screened for KO by WB**	**Confirmed KO by WB**
**Empty**	288	>30	4	0
hsKRAS.22V	288	8	8	0
hsKRAS.322	288	7	7	0

**PSN-1**	**Wells Plated**	**Grown Out**	**Screened for KO by WB**	**Confirmed KO by WB**
**Empty**	288	35	5	0
hsKRAS.138	576	66	20	0

**KP-4**	**Wells Plated**	**Grown Out**	**Screened for KO by WB**	**Confirmed KO by WB**
**Empty**	288	29	5	0
hsKRAS.138	576	50	27	**22**

**A13**	**Wells Plated**	**Grown Out**	**Screened for KO by WB**	**Confirmed KO by WB**
**Empty**	384	201	20	0
mmKras.366	384	157	60	**2**

D8	Wells Plated	Grown Out	Screened for KO by WB	Confirmed KO by WB
**Empty**	288	52	2	0
mmKras.366	288	55	10	0

E	Wells Plated	Grown Out	Screened for KO by WB	Confirmed KO by WB
mmKras.366	960	21	12	0

F	Wells Plated	Grown Out	Screened for KO by WB	Confirmed KO by WB
mmKras.366	960	106	100	0

MM1402	Wells Plated	Grown Out	Screened for KO by WB	Confirmed KO by WB
**Empty**	288	23	5	0
mmKras.138	960	87	61	**2**

MM1404	Wells Plated	Grown Out	Screened for KO by WB	Confirmed KO by WB
**Empty**	288	21	5	0
mmKras.138	960	26	21	0

PACO9	Wells Plated	Grown Out	Screened for KO by WB	Confirmed KO by WB
**Empty**	N/A	N/A	7	0
hsKRAS.138	N/A	N/A	17	**1**

PACO19	Wells Plated	Grown Out	Screened for KO by WB	Confirmed KO by WB
**Empty**	N/A	N/A	8	0
hsKRAS.138	N/A	N/A	27	**12**

Note: YAPC cells could not be plated efficiently as single cell clones following transduction with sgKRAS.

Supplementary Table 4. ICGC survival analysis

Patient ID	VITAL STATUS	DAYS TO DEATH	DAYS TO LAST FOLLOWUP	*KRAS* Genotype	Combined KO Signature Score
DO34600	1	537	NA	G12D	6223.625
DO34905	0	NA	454	G12D	6195.017
DO49204	1	156	NA	G12V	6159.762
DO34312	1	226	NA	G12D	5824.992
DO33512	1	499	NA	G12R	5579.694
DO33472	1	388	NA	G12V	5550.397
DO33168	1	8	NA	G12R	5499.965
DO33128	1	423	NA	G12D	5443.348
DO33368	1	98	NA	G12D	5348.309
DO49079	1	332	NA	G12V	5345.976
DO49080	0	NA	83	G12C	5304.615
DO32875	1	348	NA	G12D	5293.946
DO49198	1	251	NA	G12D	5267.167
DO49168	0	NA	119	G12D	5241.205
DO33480	0	NA	264	Q61H	5239.953
DO33544	1	412	NA	Q61H	5173.411
DO33336	1	451	NA	A11T	5067.42
DO49183	1	429	NA	G12D	5058.542
DO34640	1	287	NA	G12D	5054.065
DO34696	0	NA	365	G12V	4991.424
DO33376	0	NA	490	G12R	4977.434
DO49172	1	1021	NA	G12D	4976.701
DO34368	1	537	NA	G12R	4976.031
DO49138	1	230	NA	G12V	4942.775
DO49174	0	NA	838	G12D	4865.843
DO34849	1	272	NA	G12R	4823.79
DO34288	1	220	NA	G12R	4745.178
DO32878	1	260	NA	G12V	4744.607
DO34817	0	NA	268	G12R	4720.218
DO49130	0	NA	93	G12S	4593.87
DO34504	1	257	NA	-	4518.863
DO49129	0	NA	64	G12D	4516.196
DO34240	1	236	NA	G12A	4494.877
DO34736	1	203	NA	G12V	4488.779
DO32829	1	1874	NA	G12V	4442.48
DO33400	0	NA	103	G12R	4388.993
DO32860	1	1259	NA	G12D	4375.253
DO32900	1	768	NA	G12D	4359.403
DO49133	0	NA	188	G12V	4315.222
DO34432	1	400	NA	-	4230.664

DO34608	1	709	NA	G12V	4223.911
DO49090	1	152	NA	-	4169.049
DO34961	1	632	NA	-	4101.935
DO33392	0	NA	319	Q61H	4061.978
DO49164	0	NA	76	-	4023.224
DO33408	0	NA	433	G12D	3990.541
DO32863	1	715	NA	G12D	3966.621
DO34785	0	NA	525	G12R	3877.66
DO34680	1	99	NA	G12V	3824.9144
DO49137	0	NA	232	G12D	3822.34
DO49184	1	455	NA	G12R	3804.727
DO34656	0	NA	175	G12D	3751.057
DO49199	1	1095	NA	G12V	3750.558
DO49078	1	465	NA	G12V	3738.33
DO34809	1	297	NA	G12V	3735.732
DO49135	0	NA	144	G12R	3713.516
DO33552	0	NA	402	G12D	3696.484
DO49178	1	27	NA	Q61H	3660.5716
DO34793	1	427	NA	G12D	3603.0059
DO33488	0	NA	221	-	3576.963
DO34616	0	NA	295	G12V	3544.128
DO33344	1	361	NA	G12D	3527.0628
DO49076	1	480	NA	-	3469.746
DO34720	0	NA	296	G12D	3369.446
DO49113	0	NA	265	G12V	3248.7845
DO34448	1	719	NA	G12V	3117.632
DO49175	1	414	NA	G12V	3081.8074
DO34264	1	1144	NA	G12R	2982.47689
DO49087	1	260	NA	-	2959.635
DO33984	0	NA	667	G12V	2876.2697
DO49181	1	1534	NA	G12R	2875.523
DO49193	1	1	NA	G12D	2707.9262
DO34336	0	NA	224	G12R	2510.5264
DO49074	1	168	NA	Q61H	2013.4158

VITAL STATUS: 1 = died, 0 = alive at last follow-up, KRAS Genotype: “-“ genomic data not available, Note: Higher Signature Score is more similar to KO signature

Supplementary Table 5. Primers for cDNA amplification and cloning

cDNA	Template	Species	Orientation	Sequence
eGFP	*MSCV-Luciferase-IRES-GFP*	Jellyfish	Forward	ACCATGGTGAGCAAGGGCGAG
eGFP	*MSCV-Luciferase-IRES-GFP*	Jellyfish	Reverse	TTACTTGTACAGCTCGTCCATGCC
myr-HA-AKT1	*pLNCX myr HA Akt1* (Addgene #9005)	Human	Forward	ACCATGGGGTCTTCAAAATCTAAAC
myr-HA-AKT1	*pLNCX myr HA Akt1* (Addgene #9005)	Human	Reverse	TCAGGCCGTGCCGCTG
myr-HA-AKT1-K179M	*pLNCX myr HA Akt1 K179M* (Addgene #9006)	Human	Forward	ACCATGGGGAGCAGCAAGAGC
myr-HA-AKT1-K179M	*pLNCX myr HA Akt1 K179M* (Addgene #9006)	Human	Reverse	TCAGGCCGTGCCGCTG
myr-HA-AKT2	*pBabe puroL Myr HA Akt2* (Addgene #9018)	Human	Forward	ACCATGGGGAGCAGCAAGAGC
myr-HA-AKT2	*pBabe puroL Myr HA Akt2* (Addgene #9018)	Human	Reverse	TCACTCGCGGATGCTGGC
MEK-DD	*pBabe-Puro-MEK-DD* (Addgene #15268)	Mouse	Forward	ACCATGCCCAAGAAGAAGCCGAC
MEK-DD	*pBabe-Puro-MEK-DD* (Addgene #15268)	Mouse	Reverse	TCAGATGCTGGCAGCGT
KrasG12V sgRNA-resistant	*pLX304-KrasG12V*	Human	Forward	ACCATGACTGAATATAAACTTGTGGTCGTCGGTGCGGTTGGAGTAGGCAAGAG
KrasG12V	*pLX304-KrasG12V*	Human	Reverse	TTACATAATTACACACTTTGTCT
KrasG12D sgRNA-resistant Part 1	*MSCV-KrasG12D-IRES-GFP*	Mouse	Forward	ACCATGACTGAGTATAAGCTTGTGGT
KrasG12D sgRNA-resistant Part 1	*MSCV-KrasG12D-IRES-GFP*	Mouse	Reverse	TGCTAACTCCTGAGCTTGCTTGGTATCTACTGTTCTAGAAGG

KrasG12D sgRNA-resistant Part 2	*MSCV-KrasG12D-IRES-GFP*	Mouse	Forward	CCTTCTAGAACAGTAGATACCAAGCA AGCTCAGGAGTTAGCA
KrasG12D sgRNA-resistant Part 2	*MSCV-KrasG12D-IRES-GFP*	Mouse	Reverse	TCACATAACTGTACACCTTGT
Hygromycin	*MSCV-Luciferase-PGK-Hygro*	N/A	Forward	ATGAAAAAGCCTGAACTCACC
Hygromycin	*MSCV-Luciferase-PGK-Hygro*	N/A	Reverse	CTATTCCTTTGCCCTCGGAC
mCherry	*pBS-imCherry*	Coral	Forward	ACCATGGCAAGCAAGGGCGAGGAGG ATAAC
mCherry	*pBS-imCherry*	Coral	Reverese	TCAAGACTTGTACAGCTCGTCCATG

ACC Kozak sequence was added to each forward primer. sgRNA-resistant constructs were made by including silent mutations corresponding to the guide sequence. *Kras*^{G12D} sgRNA-resistant cDNA was clones as two parts joined by Gibson assembly (NEB).

Supplementary Table 6. PCR amplification primers for sequencing

Gene	Species	Exon	Orientation	Sequence
KRAS	Human	1	Forward	AAGTACAGTTCATTACGATACACGTCTGC
KRAS	Human	1	Reverse	TGTTGAGAAGAAGATAGGAAAATACTGCTG
KRAS	Human	2	Forward	TAGTGGCCATTTGTCCGTCA
KRAS	Human	2	Reverse	GCAGTCTGGAGCAAGTTACTC
KRAS	Human	3	Forward	TTTGGTGTAGTGGAAACTAGGAA
KRAS	Human	3	Reverse	CATGGACACTGGATTAAGAAGCA
KRAS	Human	4	Forward	CCTGTACACATGAAGCCATCG
KRAS	Human	4	Reverse	CACCAAAAGCCCCAAGACAG
Kras	Mouse	1	Forward	TGGCTGTTTAGATCAACAAGCTAAATGATAG
Kras	Mouse	1	Reverse	AGCCTTGGAACTAAAGGACATCACATATAA
Kras	Mouse	2	Forward	TTTGTCCACCTCCTTCTCCC
Kras	Mouse	2	Reverse	AAAGAAAGCCCTCCCCAGTT
Kras	Mouse	3	Forward	AGATGTGCCTATGGTCCTGG
Kras	Mouse	3	Reverse	AGCTGGAGTACACAGAGAGAC
SSFA2	Human	16	Forward	GCCTGGGACTTGGAGAAATG
SSFA2	Human	16	Reverse	AGTCATACGGGAGGTGGGTA
KLHDC3	Human	10	Forward	AGGTGTTCTCTGTGCTGTGA
KLHDC3	Human	10	Reverse	CTCCAGCCTTCTCCACCATA
Ncor1	Mouse	4	Forward	ACCCAGAAATGCAGGTACCA
Ncor1	Mouse	4	Reverse	CACTGCTGCCAAATGTTAGGA
Ccdc11	Mouse	6	Forward	GCTCCACTTTCATAGCCCCT
Ccdc11	Mouse	6	Reverse	CCTCTGGCCTTCTCGTCTAG

All PCR reactions were performed at 60C annealing temperature.
Forward primer for each pair was used for sequencing reaction except for exon 3 of Kras (mouse), for which reverse primer was used, and exon1 for KRAS (human) and Kras (mouse), which are as follows:

Gene	Species	Exon	Orientation	Sequence
KRAS	Human	1	Forward	CGTCTGCAGTCAACTGGAAT
Kras	Mouse	1	Reverse	CGCAGACTGTAGAGCAGCG

Supplementary Table 7. Quantitative PCR primers for copy number analysis

Gene	Species	Orientation	Sequence	Source
KRAS	Human	Forward	AAGGTGCACTGTAATAATCCAG	Modrek et al. *Mol Cancer Res*, 2009[4]
KRAS	Human	Reverse	AGACAGGTTTCTCCATCAATT	Modrek et al. *Mol Cancer Res*, 2009[4]
LINE1	Human	Forward	AAAGCCGCTCAACTACATGG	Modrek et al. *Mol Cancer Res*, 2009[4]
LINE1	Human	Reverse	TGCTTTGAATGCGTCCCAGAG	Modrek et al. *Mol Cancer Res*, 2009[4]
Kras	Mouse	Forward	TCAATGACGAACACCATCTGGA	Designed
Kras	Mouse	Reverse	TCCGTTCATTGAGACCTCAGC	Designed
Chromosome 5	Mouse	Forward	GAAGAAATTAGAGGGCATGCTTC	Sanchez-Rivera et al. *Nature*, 2014[5]
Chromosome 5	Mouse	Reverse	CTTCTCCCAGTGACCTTATGTA	Sanchez-Rivera et al. *Nature*, 2014[5]

Supplementary References

1. Ying, H. *et al.* Oncogenic kras maintains pancreatic tumors through regulation of anabolic glucose metabolism. *Cell* **149,** 656–670 (2012).
2. Viale, A. *et al.* Oncogene ablation-resistant pancreatic cancer cells depend on mitochondrial function. *Nature* **514,** 628–632 (2014).
3. Kapoor, A. *et al.* Yap1 activation enables bypass of oncogenic KRAS addiction in pancreatic cancer. *Cell* **158,** 185–197 (2014).
4. Modrek, B. *et al.* Oncogenic Activating Mutations Are Associated with Local Copy Gain. **7,** 1244–1253 (2009).
5. Sánchez-Rivera, F. J. *et al.* Rapid modelling of cooperating genetic events in cancer through somatic genome editing. *Nature* **516,** 428–431 (2014).
