# Supplementary material for: Survival of pancreatic cancer cells lacking KRAS function
Source: Nat Commun. 2017 Oct 23;8:1090. doi: 10.1038/s41467-017-00942-5 (PMC5653666; doi:10.1038/s41467-017-00942-5)
Supplement: Supplementary file 2 — Peer Review File [file 41467_2017_942_MOESM2_ESM.pdf]

Editorial Note: this manuscript has been previously reviewed at another journal that is not operating a transparent peer review scheme. This document only contains reviewer comments and rebuttal letters for versions considered at Nature Communications.
